# Stimuli‐Responsive CuFeTe_2_ Nanosheets for Amplified Cuproptosis/Ferroptosis in Triple‐Negative Breast Cancer Therapy

**DOI:** 10.1002/advs.202505739

**Published:** 2025-10-24

**Authors:** Molin Liu, Jian Zheng, Mengqi Yu, Qirui Wang, Yi Yuan, Nannan Shao, Xiaoliang Yang, Tianxi Shen, Li Wang, Aiyun Li, Rui Liu, Jimin Cao, Xi Liu, Fangfang Cao, Yanlin Feng

**Affiliations:** ^1^ Department of Cardiology, the First Hospital of Shanxi Medical University, and Key Laboratory of Cellular Physiology at Shanxi Medical University Ministry of Education Taiyuan 030001 China; ^2^ Shanxi Province Cancer Hospital Chinese Academy of Medical Sciences Cancer Hospital Affiliated to Shanxi Medical University Taiyuan 030001 China; ^3^ Key Laboratory of Big Data‐Based Precision Medicine of Ministry of Industry and Information Technology, School of Engineering Medicine Beihang University Beijing 100191 China; ^4^ CAS Key Laboratory for Nano‐Bio Interface, Suzhou Institute of Nano‐Tech and Nano‐Bionics Chinese Academy of Sciences Suzhou 215123 China; ^5^ Nanchang People's Hospital Nanchang 330000 China; ^6^ Medical Innovation Research Division Chinese PLA General Hospital Beijing 100048 China

**Keywords:** CuFeTe_2_ Nanosheets, cuproptosis, ferroptosis, NIR II mild‐photothermal effect, triple‐negative breast cancer, tumor microenvironment‐responsive

## Abstract

Triple‐negative breast cancer (TNBC) exhibits high copper and iron uptake, making cuproptosis and ferroptosis promising therapeutic strategies. However, their efficacy is limited by TNBC's intrinsic antioxidant defences. Herein, CuFeTe_2_ nanosheets (CFT) with internal tumour microenvironment (TME) responsiveness and external NIR‐II mild photothermal enhancement is developed to synergistically overcome this antioxidant defences, amplifying both pathways for improved TNBC therapy. In the acidic TME, CFT releases Fe^2+^ and Cu^2+^. Cu^2+^ reacted with glutathione (GSH) to generate Cu^+^, inhibiting glutathione peroxidase 4 (GPX4), amplifying lipid peroxidation (LPO), and triggering ferroptosis. Cu⁺ also induce dihydrolipoamide S‐acetyltransferase (DLAT) aggregation and disrupts iron‐sulfur (Fe–S) cluster proteins, initiating cuproptosis. Meanwhile, Fe^2+^ overload further reinforced ferroptosis. Both Fe^2+^ and Cu^+^ catalyze H_2_O_2_ decomposition into hydroxyl radicals (•OH), while NIR‐II photothermal effects accelerated this process, intensifying oxidative stress and ferroptosis. Moreover, ferroptosis depleted heat shock protein 70 (HSP70) and reduces ATP levels, sensitizing tumor cells to cuproptosis. The synergistic activation of ferroptosis and cuproptosis ultimately induced immunogenic cell death (ICD) and a potent immune response. Biodegraded CFT is efficiently excreted via renal filtration, ensuring high biocompatibility and safe clearance. This study presents a TME‐responsive, photothermal‐enhanced nanoplatform that effectively integrates ferroptosis and cuproptosis for potent antitumor therapy.

## Graphical abstract

Dual‐stimuli‐responsive CuFeTe_2_ nanosheets (CFT) induce cuproptosis/ferroptosis via TME‐responsive Fe^2^⁺/Cu^2^⁺ release and NIR‐II photothermal effects, amplifying ROS and depleting HSP70/ATP to trigger ICD and immune activation, while ensuring biocompatible renal clearance for TNBC therapy.

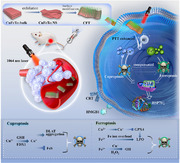

## 1 Introduction

Triple‐negative breast cancer (TNBC) is an aggressive subtype characterized by the absence of estrogen receptor (ER), progesterone receptor (PR), and human epidermal growth factor receptor 2 (HER2) overexpression, leading to high malignancy, frequent recurrence, and poor prognosis.^[^ ^1^, ^2^, ^3^ ^]^ Notably, TNBC cells exhibit elevated copper uptake and a heightened dependence on copper‐mediated signaling due to increased metabolic demands, making them particularly susceptible to cuproptosis.^[^ ^4^, ^5^, ^6^, ^7^, ^8^, ^9^, ^10^, ^11^ ^]^ Additionally, TNBC tumors are rich in iron and lipids, leading to extensive lipid peroxide (LPO) accumulation and an increased vulnerability to ferroptosis.^[^ ^12^, ^13^, ^14^ ^]^ More importantly, ferroptosis complements cuproptosis by downregulating heat shock protein 70 (HSP70), a key regulator of proteotoxic stress.^[^ ^15^, ^16^ ^]^ Despite their therapeutic potential, both cuproptosis and ferroptosis are significantly hindered by intrinsic antioxidant defenses.^[^ ^10^, ^17^ ^]^ For instance, cellular copper homeostasis mechanisms tightly regulate Cu levels, while high glutathione (GSH) levels in the tumor microenvironment (TME) chelate Cu ions and support glutathione peroxidase 4 (GPX4) in detoxifying LPO. Additionally, ATP‐dependent transporters actively expel excess Cu, further limiting cuproptosis. To overcome these barriers, we propose that depleting GSH while inhibiting intracellular ATP production could enhance the therapeutic efficacy of cuproptosis and ferroptosis in TNBC.

GSH depletion can be achieved by disrupting intracellular redox balance‐boosting reactive oxygen species (ROS) generation while simultaneously suppressing antioxidant defenses. Recently, nanonaterials incorporating multivalent metal ions (e.g., Fe^2+^/^3+^, Cu^1+^/^2+^) have shown promise in achieving this goal. They can respond to TME, catalyzing the conversion of excess hydrogen peroxide (H_2_O_2_) in the TME into toxic hydroxyl radicals (•OH), while simultaneously depleting GSH through redox reactions, thereby further weakening antioxidant defences. Moreover, they could exhibit strong absorption in the near‐infrared II (NIR II) region, enabling NIR‐II‐triggered mild localized hyperthermia, which further enhances catalytic ROS production to improve antitumor activity and effectively inhibits intracellular ATP.^[^ ^18^, ^19^ ^]^ Hence, it is highly desirable to develop an all‐in‐one nanoplatform with mild photothermal promoted GSH depletion and ATP inhibition for effectively combating TNBC cells. However, existing multivalent metal ion nanoplatforms are limited and often rely on multi‐component assemblies, such as multivalent metal ion carriers, photosensitizers, and O_2_‐supplementing agents, which increases complexity and makes the activity dependent on the ratio between these components.^[^ ^10^, ^20^, ^21^, ^22^, ^23^ ^]^ Therefore, developing a simple all‐in‐one nanoplatform with mild photothermal‐enhanced GSH depletion and ATP inhibition presents an effective strategy for combating TNBC.

**Figure 1:**
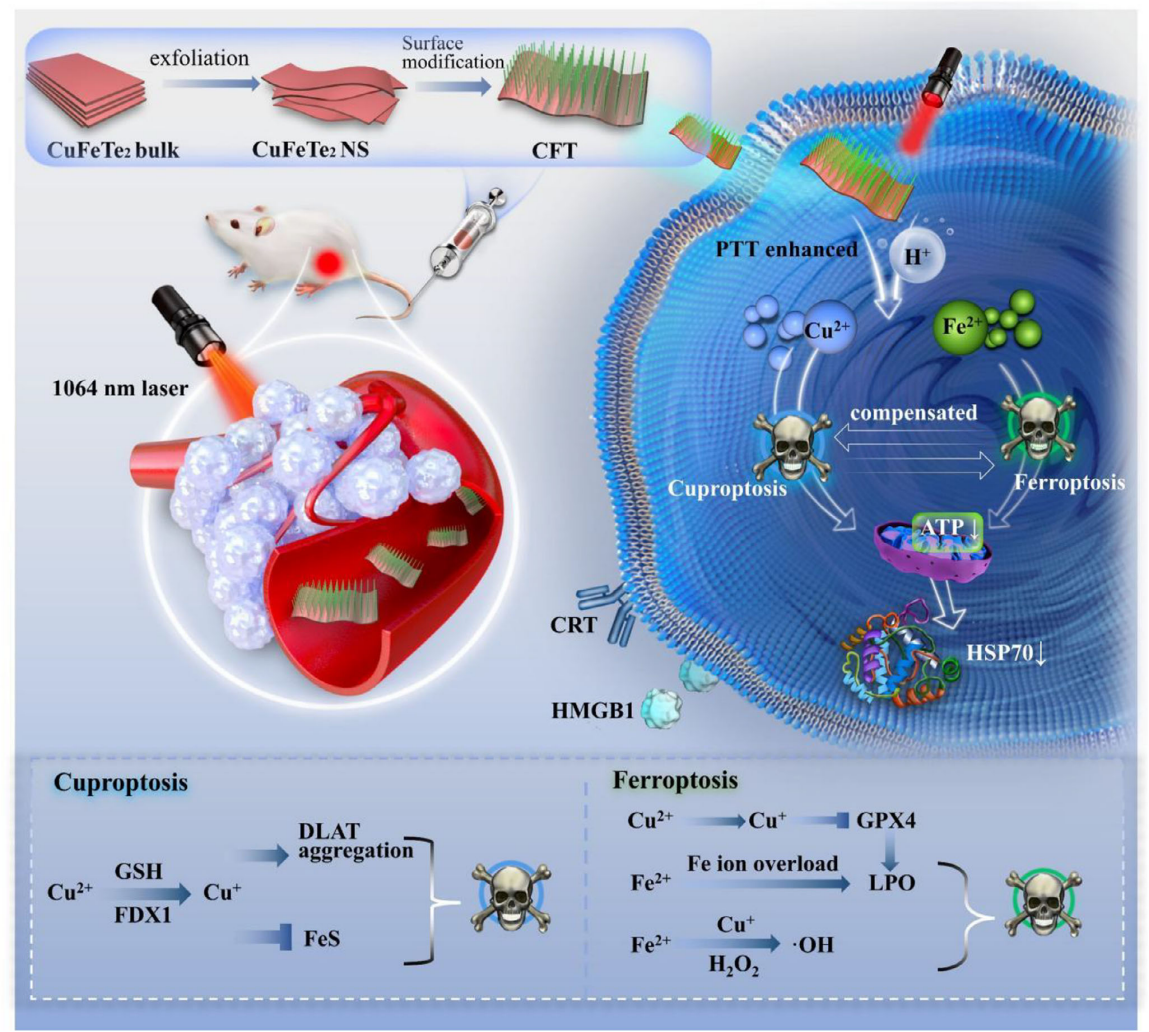
Schematic illustration of the synthesis process of CFT A) and the dual‐stimuli‐responsive CFT enabling amplified ferroptosis/cuproptosis for TNBC therapy B).

To meet this demand, we developed a dual‐stimuli‐responsive CuFeTe_2_ nanosheet (CFT) that respond to both the internally TME and external NIR II radiation, promoting GSH depletion and ATP inhibition, and thereby effectively amplifying ferroptosis and cuproptosis against TNBC (**Figure** **1**). Notably, the introduction of tellurium (Te) in this work is mainly attributed to two reasons. First, Te was incorporated to construct CuFeTe_2_ nanosheets, thereby enhancing photothermal performance^[^ ^24^, ^25^, ^26^, ^27^ ^]^ and accelerating catalytic activity. Second, as a group VI‐A element analogous to S and Se, Te exhibits unique biochemical reactivity toward thiol‐containing biomolecules to promot GSH depletion,^[^ ^28^, ^29^, ^30^, ^31^ ^]^ which strengthens ferroptosis. Overall, the resulting CuFeTe_2_ nanosheets display strong absorption in the NIR‐II region, enabling efficient photothermal conversion with deeper tissue penetration and improved biosafety. Upon NIR‐II irradiation, the local temperature rapidly increases to ≈43 °C, further accelerating catalytic reactions and potentiating ferroptosis. Compared with monometallic Cu_2‐x_Te nanomaterials,^[^ ^32^, ^33^ ^]^ CFT offers distinct advantages through Fe incorporation. Once internalized by TNBC cells, CFT could degrade to release Fe^2+^ and Cu^2+^, with Cu^2+^ being reduced to Cu^+^ by FDX1 and GSH in mitochondria. This process triggered S‐acetyltransferase (DLAT) oligomerization and iron‐sulfur (Fe−S) cluster interference, resulting in cuproptosis. Meanwhile, GSH depletion inactivated GPX4, while Fe^2+^ accumulation induced ferroptosis via lethal lipid peroxidation (LPO). Additionally, Fe^2+^ and Cu^+^ further catalyze H_2_O_2_ decomposition through a peroxidase (POD)‐like reaction, amplifying ROS production. The resulting ROS and LPO depleted HSP70 and lowered ATP levels, sensitizing tumors to cuproptosis, reducing Cu^2+^ efflux, and promoting high mobility group box1 (HMGB1) release, which induced immunogenic cell death (ICD) and a potent immune response. The dynamic redox cycling between Cu^2+^/Cu^+^ and Fe^3+^/Fe^2+^ sustains oxidative stress amplification, enabling CFT nanosheets to simultaneously exploit Fe‐ and Cu‐dependent cell death mechanisms while leveraging superior NIR‐II optical properties for enhanced TNBC therapy.

## 2 Results and Discussion

### 2.1 Preparation and Characterization of CFT

**Figure 2:**
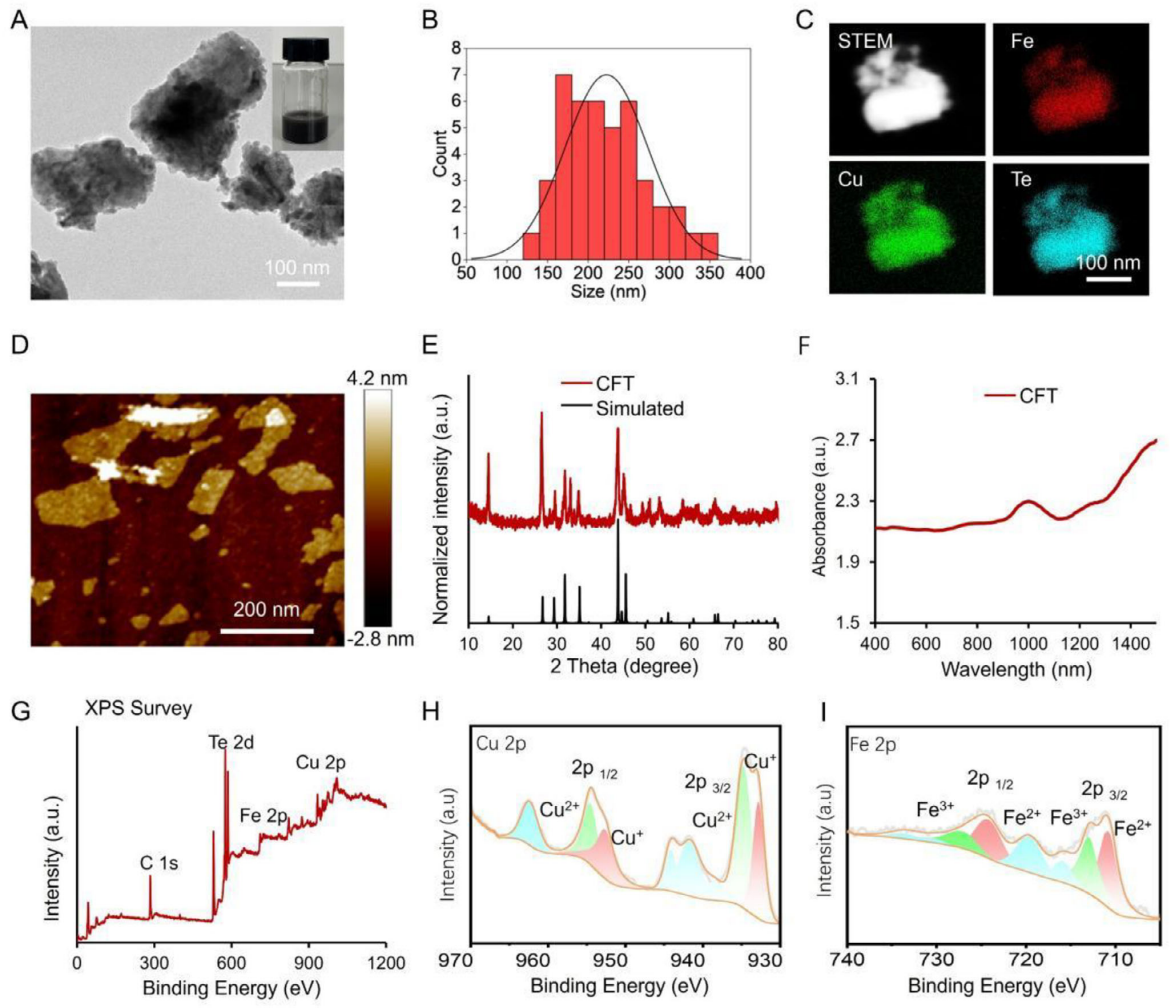
Characterization of the synthesized CFT. A) TEM images of CFT and their dispersion in deionized water (5 mg mL^−1^). B) Size distribution of CFT in Figure 2A. C) STEM with corresponding EDS elemental mapping images, D) AFM images, E) XRD pattern, F) UV–vis‐NIR absorption spectra, G) XPS spectrum, H) high‐resolution Cu 2p spectra and I) high‐resolution Fe 2p spectra of CFT.

CFT were synthesized via sonication‐assisted liquid delamination of bulk CuFeTe_2_ crystals, followed by PEG modification. Transmission electron microscopy (TEM) images revealed the characteristic sheet‐like morphology of the CFT, with an average lateral size of 222.6 nm (**Figure** **2**A,B). Moreover, it exhibited uniform dispersion in deionized water, as depicted in Figure 2A (inset). Scanning transmission electron microscopy dark field (STEM‐DF) images and the corresponding energy‐dispersive X‐ray spectroscopy (EDX) elemental mapping demonstrated the uniform distribution of Cu, Fe, and Te components throughout the nanosheets (Figure 2C; Figure S1, Supporting Information). Atomic force microscopy (AFM) further validated the freestanding morphology of CFT, with a measured thickness of approximately 2.2 nm (Figure 2D; Figure S2, Supporting Information). X‐ray powder diffraction (XRD) patterns analysis confirmed the consistency of consistent crystal structure, in agreement with simulated results (Figure 2E).^[^ ^34^ ^]^ The elemental composition of the CFT nanosheets was determined by inductively coupled plasma mass spectrometry (ICP‐MS), revealing Cu, Fe and Te contents of 24.6, 5.3, and 13.1 wt%, respectively. The ultraviolet‐visible‐near‐infrared (UV–vis‐NIR) absorbance spectrum exhibited a broad absorption band in the NIR II, analogous to traditional 2D nanosheets,^[^ ^35^, ^36^ ^]^ positioning CFT as promising candidates for photothermal therapeutic applications (Figure 2F). The X‐ray photoelectron spectroscopy (XPS) survey spectra further corroborated the presence of Cu, Fe, and Te elements in the CFT (Figure 2G), consistent with the EDX analysis presented in Figure S1 (Supporting Information). The high‐resolution spectra of Cu 2p and Fe 2p disclosed tha Cu was present in both Cu^2+^ (954.17 and 934.48 eV) and Cu^+^ (952.28 and 932.68 eV) oxidation states, with a Cu^2+^/Cu^+^ ratio of approximately 1.5, while Fe was identified in both Fe^2+^ (723.77 and 710.7 eV) and Fe^3+^ (726.1 and 712.7 eV) states, with a Fe^2+^/Fe^3+^ ratio of approximately 1.0 (Figure 2H,I). These valence states are crucial for facilitating the Fenton reaction mediated by Cu^+^ and Fe^2+^, as well as the consumption of GSH by Cu^2+^ and Fe^3+^. Dynamic light scattering (DLS) measurements indicated an increase in hydrodynamic size from 280.0 ± 14.95 nm to 330.4 ± 3.12 nm with narrow polydispersity index (PDI) (Figure S3A, B; Figure S4A, Supporting Information), coupled with a decrease in zeta potential from ‐8.574 ± 0.38 to ‐11.175 ± 0.6, confirming the successful PEG coating on the surface of the CFT (Figure S3C, Supporting Information). Furthermore, thermalgravimetric analysis (TGA) determined the PEG content to be 6.04% by mass (Figure S5, Supporting Information). The colloidal stability of CFT was systematically evaluated in different media, including H_2_O, Dulbecco's modified Eagle medium (DMEM), and DMEM containing 10% fetal bovine serum (FBS). DLS measurements revealed that CFT maintained uniform hydrodynamic sizes and acceptable PDI values in all tested media even after six months of storage, indicating excellent colloidal stability (Figure S4A—F, Supporting Information). Furthermore, CFT dispersions across a wide concentration range (10, 50, 100, 1000 µg mL^−1^) exhibited consistent hydrodynamic sizes and PDI values, demonstrating robust resistance to aggregation upon dilution (Figure S6, Supporting Information). Collectively, these results confirm that CFT possesses favorable stability, a prerequisite for reliable biomedical applications.

### 2.2 Performance of CFT

**Figure 3:**
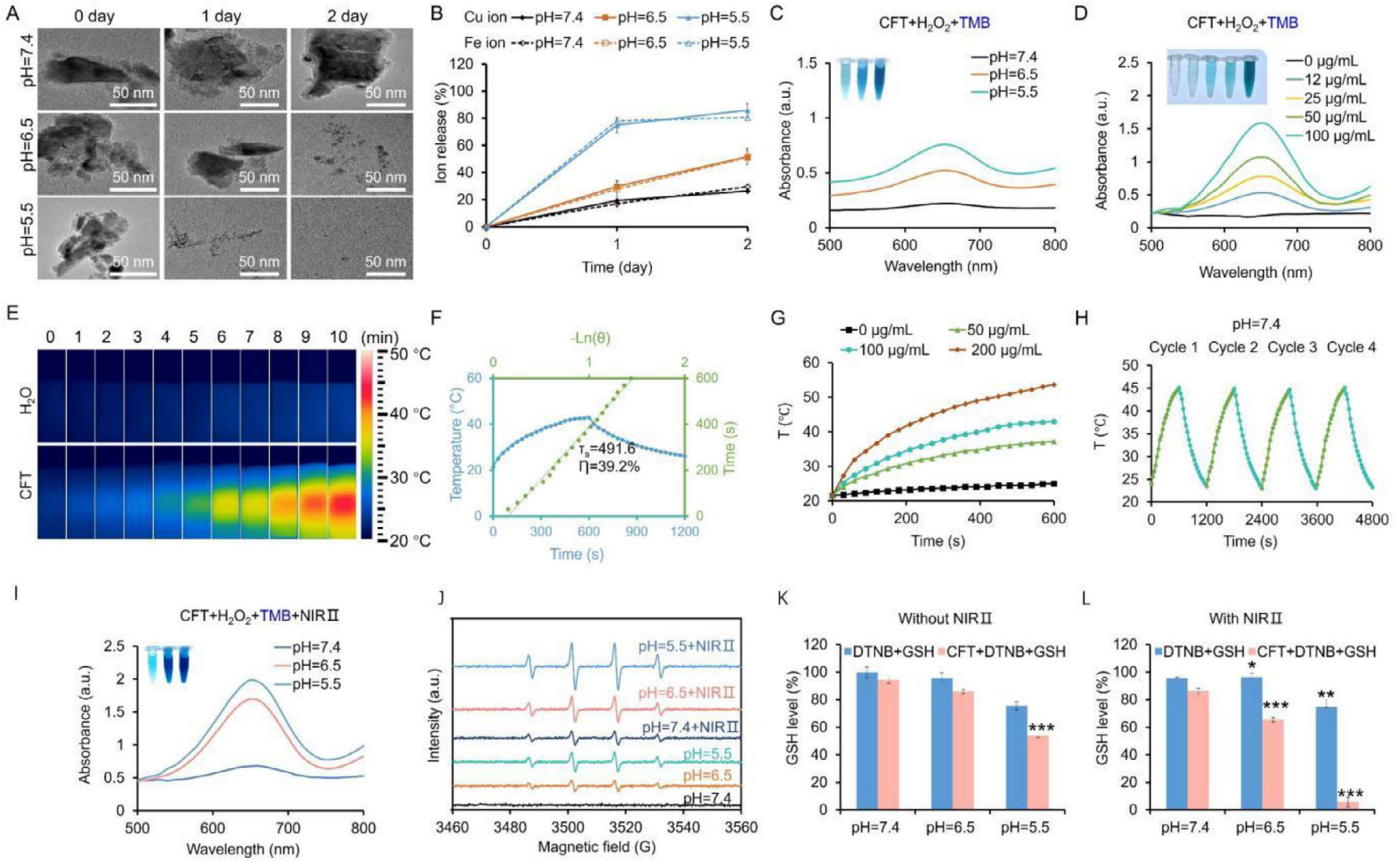
Performance of CFT. A) TEM images of CFT in PBS under different pH conditions at day 0, 1 and 2. B) Release profiles of Cu and Fe ions at varying pH values. UV–vis absorption spectra of oxidized TMB under different pH conditions C) and CFT concentrations D) in the presence of H_2_O_2_ and CFT. E) Infrared thermal images of CFT (100 µg mL^−1^) under 1064 nm laser irradiation (1 W cm^−2^) for 10 min. F) Photothermal heating curve of CFT (100 µg mL^−1^) under 1064 nm laser irradiation (1 W cm^−2^) and corresponding cooling curve. The green line represents the linear fit of −lnθ versus time, obtained from the cooling curve. G) Photothermal heating curves of CFT at different concentrations under 1064 nm laser irradiation (1 W cm^−2^) for 10 min. H) Photothermal heating and natural cooling cycles of CFT at pH = 7.4. I) UV–vis absorption spectra of oxidized TMB recorded under different pH conditions values in the presence of H_2_O_2_ and CFT with 1064 nm laser irradiation (1 W cm^−2^, 10 min). J) DMPO spin‐trapping ESR spectra of CFT under different pH conditions values with or without 1064 nm laser irradiation in the presence of H_2_O_2_ and CFT. Detection of GSH levels in the absence K) and presence L) of 1064 nm laser irradiation (1 W cm^−^^2^, 10 min). Control groups without CFT were included to assess the effect of acidic pH and laser irradiation on GSH levels. *p < 0.05, ** *p* < 0.01, *** *p* < 0.001.

Due to the acidic nature of TME, we first evaluated the stability of CFT in solutions with varying pH levels before proceeding with further applications. TEM imaging revealed that CFT maintained its structural integrity under neutral conditions; however, its two‐dimensional architecture gradually degraded over time under acidic conditions (pH = 6.5 and pH = 5.5, **Figure** **3**A). Moreover, the ICP‐MS analysis confirmed a pH‐dependent release of Cu and Fe ions, with significantly accelerated ion dissolution observed under acidic conditions, indicating the pH‐responsive degradability of CFT (Figure 3B). This intrinsic biodegradability facilitates the metabolic clearance of CFT from the body, thereby mitigating potential long‐term toxicity and enhancing biocompatibility.

The ROS‐generating capability of CFT under different pH conditions was subsequently evaluated using 3,3′,5,5′ tetramethylbenzidine (TMB) colorimetric assays, wherein •OH catalyze the oxidation of TMB to its blue oxidized form (oxTMB) with absorption peak at 652 nm. As shown in Figure 3C, the absorbance peak of oxTMB at 652 nm increased as the pH decreased, indicating enhanced •OH production. To further validate •OH generation, we performed electron spin resonance (ESR) measurements using DMPO as the spin‐trapping agent. As demonstrated in Figure 3J, a characteristic 1:2:2:1 ESR signal of DMPO/•OH was observed, which was more pronounced under acidic conditions, consistent with the TMB assay results. Notably, in the absence of H_2_O_2_, the CFT + TMB group exhibited negligible absorbance at 652 nm and no detectable ESR signal, demonstrating that H_2_O_2_ is indispensable for initiating the peroxidase‐like catalytic activity (Figure S7A, B, Supporting Information). Furthermore, the oxTMB absorbance exhibited a concentration‐dependent increase when the CFT concentration was raised from 0 to 100 µg mL^−1^ (Figure 3D). Given that mild photothermal heating can enhance nanocatalytic therapeutic efficacy^[^ ^37^, ^38^ ^]^ and that CFT exhibits strong absorption in the NIR II region, we further investigated its PTT and ROS‐generating performance under 1064 nm irradiation. Infrared thermal imaging revealed a significant temperature increase from 20 °C to 43 °C after 10 minutes of irradiation (1.0 W cm^−2^) at 100 µg mL^−1^, achieving localized mild hyperthermia, typically maintained within the 40–45 °C range (Figure 3E). Based on the recorded temperature elevation profile, the photothermal conversion efficiency (η) of CFT was calculated using Roper's method^[^ ^39^ ^]^ and determined to be 39.2% (Figure 3F). Additionally, real‐time temperature monitoring demonstrated that the thermal response of CFT was both concentration‐ and power density‐dependent (Figure 3G; Figure S8, Supporting Information). Photostability and photothermal conversion efficiency (η) are critical factors influencing the photothermal and therapeutic performance of nanomaterials. The photostability of CFT was assessed by recording temperature variations over four laser irradiation/natural cooling cycles at different pH values. Heating curves displayed comparable temperature elevations across all tested pH conditions, and the repeated on/off cycles confirmed stable photothermal performance even in acidic environments (Figure 3H; Figure S9A, B, Supporting Information). TEM and DLS analyses further demonstrated that 10 min of 1064 nm laser irradiation caused no detectable morphological changes or size reduction at either pH, indicating negligible structural degradation during the irradiation period (Figure S10, Supporting Information).

To investigate whether the localized mild hyperthermia generated by CFT could further accelerate the Fenton‐like reaction, we evaluated •OH production under 1064 nm NIR‐II laser irradiation. As shown in Figure 3I and J, laser exposure markedly enhanced oxTMB absorbance and intensified the characteristic DMPO/•OH ESR signals at both neutral and acidic pH, compared with non‐irradiated controls. Furthermore, a concentration‐dependent increase in •OH generation was observed under irradiation, as evidenced by the progressive rise in oxTMB absorbance with increasing CFT concentrations (Figure S11, Supporting Information). These results demonstrate that photothermal activation synergizes with the intrinsic pH‐dependent peroxidase‐like activity of CFT, leading to more efficient ROS production. Additionally, the presence of high‐valence Cu^2+^ and Fe^3+^ enabled the depletion of excessive endogenous GSH within the TME. The GSH‐depleting capability of CFT was evaluated using a 5,5′‐dithiobis‐(2‐nitrobenzoic acid) (DTNB) assay, in which GSH react with DTNB to yield the yellow chromophore 2‐nitro‐5‐thiobenzoate (TNB^2−^) with a characteristic absorbance at 412 nm. As shown in Figure 3K, CFT induced a pH‐dependent GSH consumption, with markedly greater depletion observed under acidic conditions. To exclude potential interference from protonation of TNB^2−^ at low pH, control experiments without CFT were conducted and observed that acidic pH alone caused a slight decrease in absorbance. However, CFT‐treated groups exhibited significantly greater GSH depletion than pH‐only controls, confirming that the effect originated from CFT‐mediated catalytic activity. Furthermore, upon exposure to 1064 nm laser irradiation, GSH depletion was further enhanced in the presence of CFT (Figure 3L), and NIR‐II irradiation alone had negligible influence on GSH levels, indicating that mild photothermal effects enhanced the catalytic GSH consumption capability of CFT. Furthermore, the dynamic redox cycling between Cu^2+^/Cu^+^ and Fe^3+^/Fe^2+^ facilitated the continuous regeneration of catalytically active species, thereby sustaining the oxidative stress amplification necessary for enhanced tumour therapeutic efficacy.

### 2.3 In Vitro Therapeutic Effect of CFT

**Figure 4:**
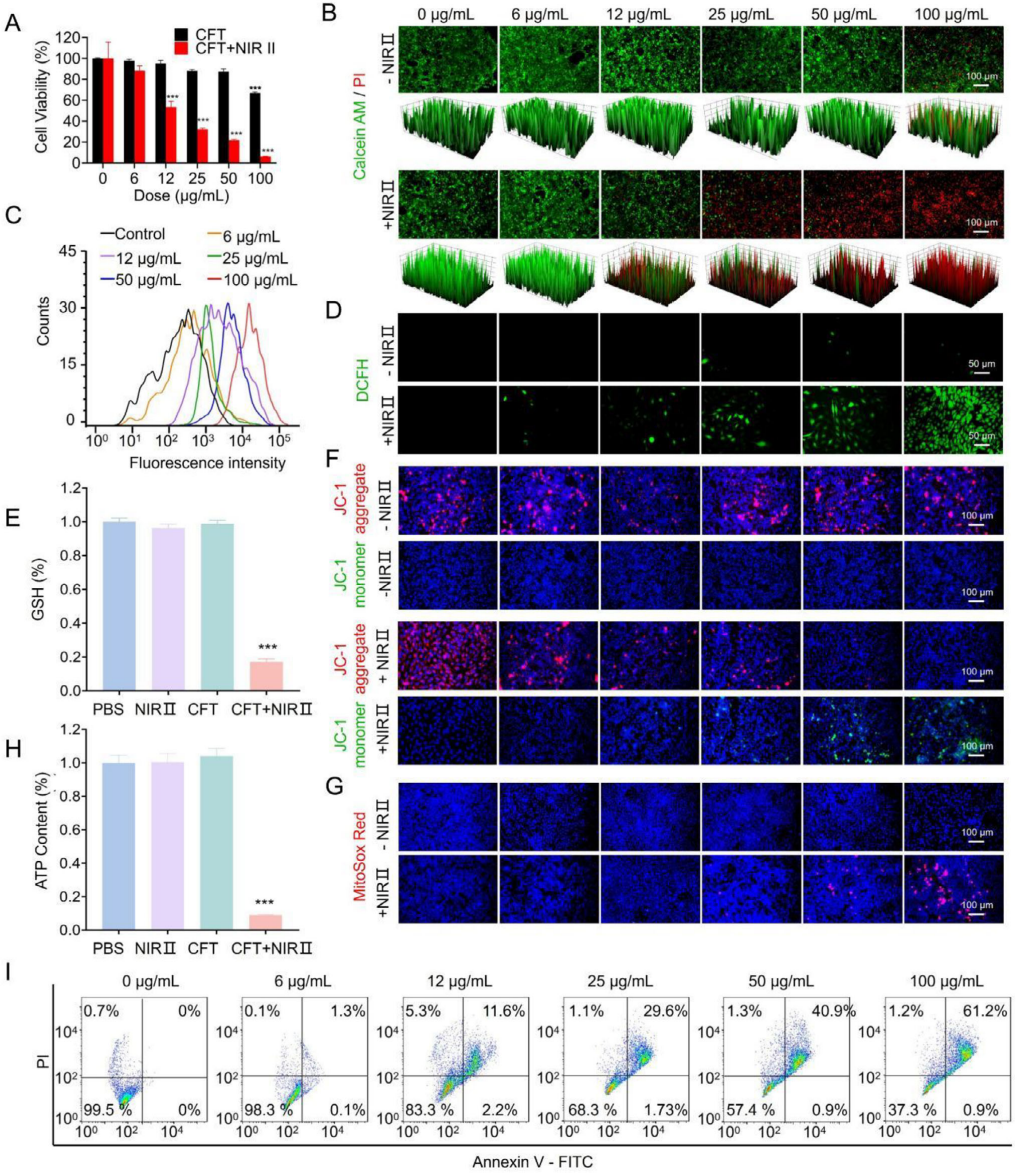
In vitro therapeutic effect of CFT. A) CCK‐8 assay of 4T1 cell viability following treatment with varying concentrations of CFT for 24 h, with or without 1064 nm laser irradiation (1 W cm^−2^, 10 min) (n = 3). B) Live/dead staining of 4T1 cells treated with 0–100 µg mL^−1^ CFT for 24 h with or without 1064 nm laser irradiation (1 W cm^−2^, 10 min). C) Flow cytometric analysis of intracellular ROS levels under 1064 nm laser irradiation (1 W cm^−2^, 10 min). D) Fluorescence imaging of DCFH‐DA stained cells, with or without 1064 nm laser irradiation (1 W cm^−2^, 10 min). E) Quantification of intracellular GSH levels using the DTNB assay (n = 3). F) Mitochondrial membrane potential assessment via JC‐1 staining, with or without 1064 nm laser irradiation (1 W cm^−2^, 10 min). G) Mitochondrial superoxide generation analysis using MitoSox Red staining with or without 1064 nm laser irradiation (1 W cm^−2^, 10 min). Cell nuclei were stained with Hoechst 33342 (blue). H) Measurement of cellular ATP content (n = 3). I) Flow cytometric analysis of apoptotic cells following 1064 nm laser irradiation (1 W cm^−2^, 10 min). *** *p* < 0.001.

The remarkable ROS generation and GSH depletion capabilities, coupled with the in‐situ photothermal effect of CFT, underscore their potential therapeutic efficacy in cancer treatment. The cytotoxicity of CFT on different cells was assessed by the cell counting kit‐8 (CCK‐8) assay. As shown in Figure S12A—C (Supporting Information), all normal cells (mouse embryonic fibroblast (NIH‐3T3) cells, human embryonic kidney 293T (HEK 293T) cells, and murine monocyte‐macrophage leukemia (RAW 264.7) cells) maintained high viability even at 100 µg mL^−1^, demonstrating favorable biocompatibility. In contrast, the murine mammary carcinoma 4T1 cells exhibited a concentration‐dependent decrease in viability, with 67% survival at 100 µg mL^−1^ (**Figure** **4**A), suggesting preferential cytotoxicity toward tumor cells. This selectivity may be attributed to the mildly acidic TME,^[^ ^40^, ^41^, ^42^, ^43^, ^44^ ^]^ which facilitates CFT degradation and enhances peroxidase‐like catalytic activity, thereby accelerating ROS generation. To further confirm this phenomenon, two additional tumor cell lines (MDA‐MB‐231 human breast cancer cells and B16‐F10 murine melanoma cells) were tested and displayed similar trends to that in 4T1 cells toward CFT (Figure S13, Supporting Information). Moreover, when the culture medium was adjusted to pH 6.5 to mimic the TME, all tested cells—including both tumor and normal cells—exhibited significantly enhanced cytotoxicity (Figure S14, Supporting Information), indicating that acidity is the key factor triggering CFT‐induced toxicity. Collectively, these findings demonstrate that the apparent selectivity of CFT arises from the intrinsic acidic TME of tumor cells, while normal cells remain largely unaffected under physiological pH. Building on this selective cytotoxicity, we next investigated the synergistic phototherapeutic potential of CFT under NIR‐II irradiation. Upon exposure to 1064 nm laser irradiation, a concentration‐dependent reduction in cell viability was observed, with approximately 90% of 4T1 cells undergoing cell death at a CFT concentration of 100 µg mL^−1^. Live/dead cell staining and corresponding 3D surface plots (Figure 4B) further validated the potent phototherapeutic effects of CFT. To elucidate the underlying mechanisms, intracellular ROS generation was analyzed using the DCF assay and evaluated by flow cytometry (Figure 4C; Figure S15, Supporting Information) and fluorescence imaging (Figure 4D). In the absence of laser irradiation, no detectable DCF fluorescence was observed in 4T1 cells. However, under 1064 nm laser exposure, green fluorescence intensity increased in a dose‐dependent manner, confirming enhanced ROS production. Furthermore, GSH depletion in 4T1 cells following CFT treatment was quantified using the DTNB assay. As depicted in Figure 4E, cells treated with CFT under 1064 nm laser irradiation exhibited significantly reduced GSH levels compared to those treated with either CFT alone or laser irradiation alone, highlighting the effective GSH depletion.

The combined effects of ROS generation and GSH depletion exacerbated oxidative stress, leading to mitochondrial dysfunction, ATP depletion, and ultimately apoptosis. Mitochondrial dysfunction was further characterized using JC‐1 and MitoSox Red staining to assess mitochondrial membrane potential (ΔΨm) and superoxide generation, respectively. Elevated oxidative stress can induce mitochondrial membrane depolarization and excessive mitochondrial superoxide accumulation. JC‐1 staining revealed that, in untreated or non‐irradiated cells, mitochondria maintained a high ΔΨm, as evidenced by dominant red fluorescence. However, following 1064 nm laser irradiation, cells exhibited a pronounced reduction in red fluorescence and an increase in green fluorescence, indicating mitochondrial membrane depolarization in a concentration‐dependent manner (Figure 4F). Quantitative analysis further confirmed a dose‐dependent decrease in the red‐to‐green fluorescence ratio (Figure S16, Supporting Information), demonstrating that CFT effectively disrupted ΔΨm. Mitochondrial depolarization is associated with excessive mitochondrial superoxide production, which was assessed via MitoSox Red staining. In the absence of laser irradiation, negligible red fluorescence was detected (Figure 4G). However, upon 1064 nm laser irradiation, red fluorescence intensity progressively increased with higher CFT concentrations, confirming mitochondrial superoxide accumulation and oxidative stress‐induced damage (Figure 4G; Figure S17, Supporting Information). Consistently, ATP levels were significantly depleted in cells treated with CFT and 1064 nm laser irradiation, further corroborating severe mitochondrial impairment (Figure 4H). To determine whether mitochondrial dysfunction culminated in apoptosis, Annexin V‐FITC (AV)/propidium iodide (PI) staining was performed. As shown in Figure 4I, following 6 h of incubation with CFT and 1064 nm laser irradiation, apoptosis was observed in approximately 61% of 4T1 cells at a concentration of 100 µg mL^−1^. Collectively, these findings demonstrate that CFT, upon NIR II irradiation, effectively induce apoptosis in tumor cells through an intrinsic mitochondria‐mediated apoptotic pathway, highlighting their potential as a promising therapeutic strategy for cancer treatment.

### 2.4 In Vitro Cuproptosis and Ferroptosis Evaluation

**Figure 5:**
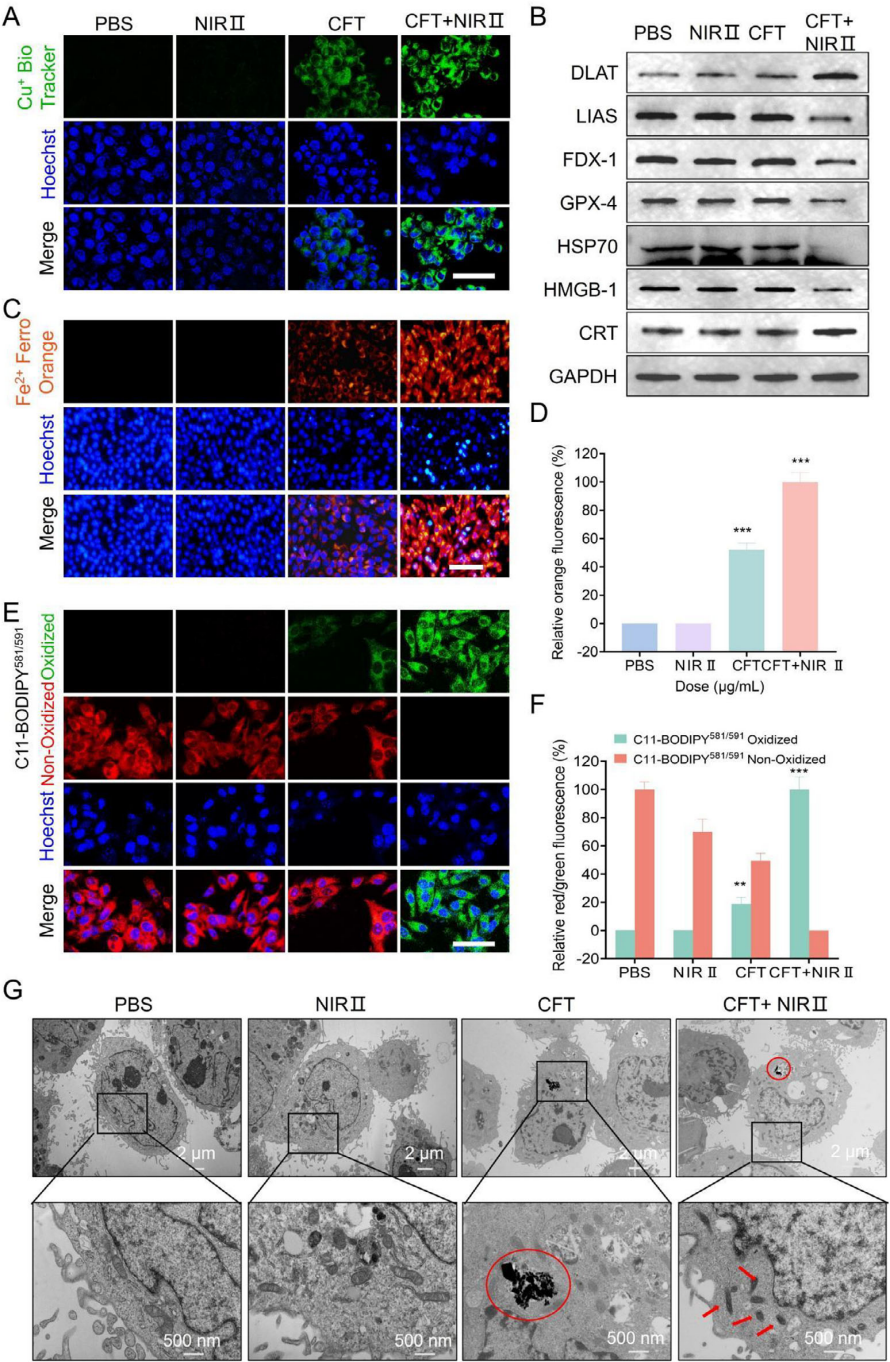
In vitro evaluation of cuproptosis and ferroptosis. A) CLSM images of 4T1 cells stained with Cu^+^ BioTracker after different treatments. Scale bar: 50 µm. B) Western blot analysis of HSP‐70, DLAT, LIAS, FDX‐1, GPX‐4, CRT, and HMGB‐1 expression levels. The corresponding uncropped western blots and intensity analysis are provided in Figure S19 (Supporting Information). C) Fluorescence images of FerroOrange‐stained cells following various treatments, with corresponding semi‐quantitative analysis shown in D). Scale bar: 100 µm. E) CLSM images of 4T1 cells stained with C11‐BODIPY^581/591^ after different treatments, with the corresponding semi‐quantitative analysis presented in F). Scale bar: 50 µm. G) Bio‐TEM images of 4T1 cells subjected to different treatments. Red circles highlight the presence of CFT, while red arrows indicate reduced or absent mitochondrial cristae. **p < 0.01, ***p < 0.001.

Encouraged by the augmented intracellular oxidative stress, we further investigated the cuproptosis and ferroptosis performance of CFT. Cuproptosis is initiated by excessive intracellular Cu^2+^ accumulation, which is reduced by FDX1 to the more toxic Cu^+^ form. To assess intracellular Cu^+^ levels, we utilized Cu^+^ BioTracker. As shown in **Figure** **5**A and Figure S18 (Supporting Information), CFT effectively released Cu^+^, with a notably higher Cu^+^ content observed upon 1064 nm irradiation. This enhancement may be attributed to mild hyperthermia‐augmented cellular membrane fluidity and thermally accelerated Fenton‐like reactions.^[^ ^45^, ^46^, ^47^, ^48^ ^]^ Elevated Cu^+^ levels promote dihydrolipoamide S‐acetyltransferase (DLAT) aggregation and disrupt iron–sulfur (Fe–S) cluster proteins, thereby initiating cuproptosis.^[^ ^49^ ^]^ Consistently, western blot (WB) analysis revealed decreased expression of FDX1 and lipoyl synthase (LIAS, a Fe–S cluster protein), together with increased DLAT levels in the CFT + NIR‐II group, confirming the activation of cuproptosis (Figure 5B; Figure S19A—C, Supporting Information).

Similarly, ferroptosis is characterized by Fe^2+^ overload. We employed FerroOrange to detect intracellular Fe^2+^ levels, revealing significant Fe^2+^ accumulation in 4T1 cells treated with CFT + NIR II compared to CFT alone (Figure 5C,D). The biodegradation of CFT contributed to Fe^2+^ release, expanding intracellular iron pools and triggering ferroptosis. To further validate ferroptosis induction, we examined key biomarkers, including intracellular LPO, GSH levels, GPX4 expression, and mitochondrial morphology. A hallmark of ferroptosis is lipid peroxidation (LPO), driven by Fe^2+^‐mediated Fenton reactions that generate ROS via multiple pathways. LPO levels were evaluated using the C11‐BODIPY^581/591^ fluorescence probe. Notably, the attenuation of red fluorescence and the corresponding enhancement of green fluorescence (oxidized C11‐BODIPY^581/591^) in the CFT + NIR II group indicated effective LPO accumulation (Figure 5E,F). As a key antioxidant system, the GPX4/GSH axis mitigates LPO to suppress ferroptosis. Accordingly, increasing intracellular ROS production while inactivating GPX4 represents a crucial strategy to promote ferroptosis. The CFT + NIR II group significantly enhanced ROS generation and GSH depletion (Figure 4D,E), further corroborating its ferroptosis‐inducing potential. Moreover, GPX4 expression was markedly reduced in the CFT + NIR II group compared to other treatments (Figure 5B; Figure S19D, Supporting Information). These findings suggest that CFT, with its potent GSH‐depleting and ROS‐generating capabilities, effectively disrupts the GSH/GPX4 axis, facilitates LPO formation, and ultimately triggers ferroptosis in 4T1 cells.

Furthermore, mild hyperthermia‐induced LPO accumulation and ATP depletion contributed to stress‐induced cleavage of heat shock proteins (HSP70), compromising the protective mechanisms of cancer cells and further sensitizing them to cuproptosis (Figure 5B; Figure S19E, Supporting Information). Bio‐TEM imaging revealed severe mitochondrial damage in 4T1 cells treated with CFT + NIR II, as evidenced by increased membrane density, reduced mitochondrial volume, and diminished or absent cristae—hallmarks of both cuproptosis and ferroptosis (Figure 5G). Beyond directly inducing cell death, cuproptosis and ferroptosis can also elicit a potent immune response by triggering immunogenic cell death (ICD). This occurs through the release of key signaling molecules, such as calreticulin (CRT) and high‐mobility group box 1 (HMGB1), which serve as critical mediators of immune activation. CRT exposure on the cell surface acts as an “eat me” signal, facilitating dendritic cell (DC) recognition and maturation. As shown in Figure 5B and Figure S19F (Supporting Information), CRT expression was significantly upregulated on the surface of 4T1 cells following CFT + NIR II treatment. Additionally, HMGB1, which functions as a “present me” signal to enhance ICD, was released upon CFT + NIR II exposure, as evidenced by its decreased intracellular expression (Figure 5B; Figure S19G, Supporting Information). These findings highlight the dual therapeutic potential of CFT + NIR II, not only in inducing direct cancer cell death but also in amplifying the immune response through ICD, ultimately enhancing tumour immunogenicity.

### 2.5 Anticancer Biological Mechanism of CFT

**Figure 6:**
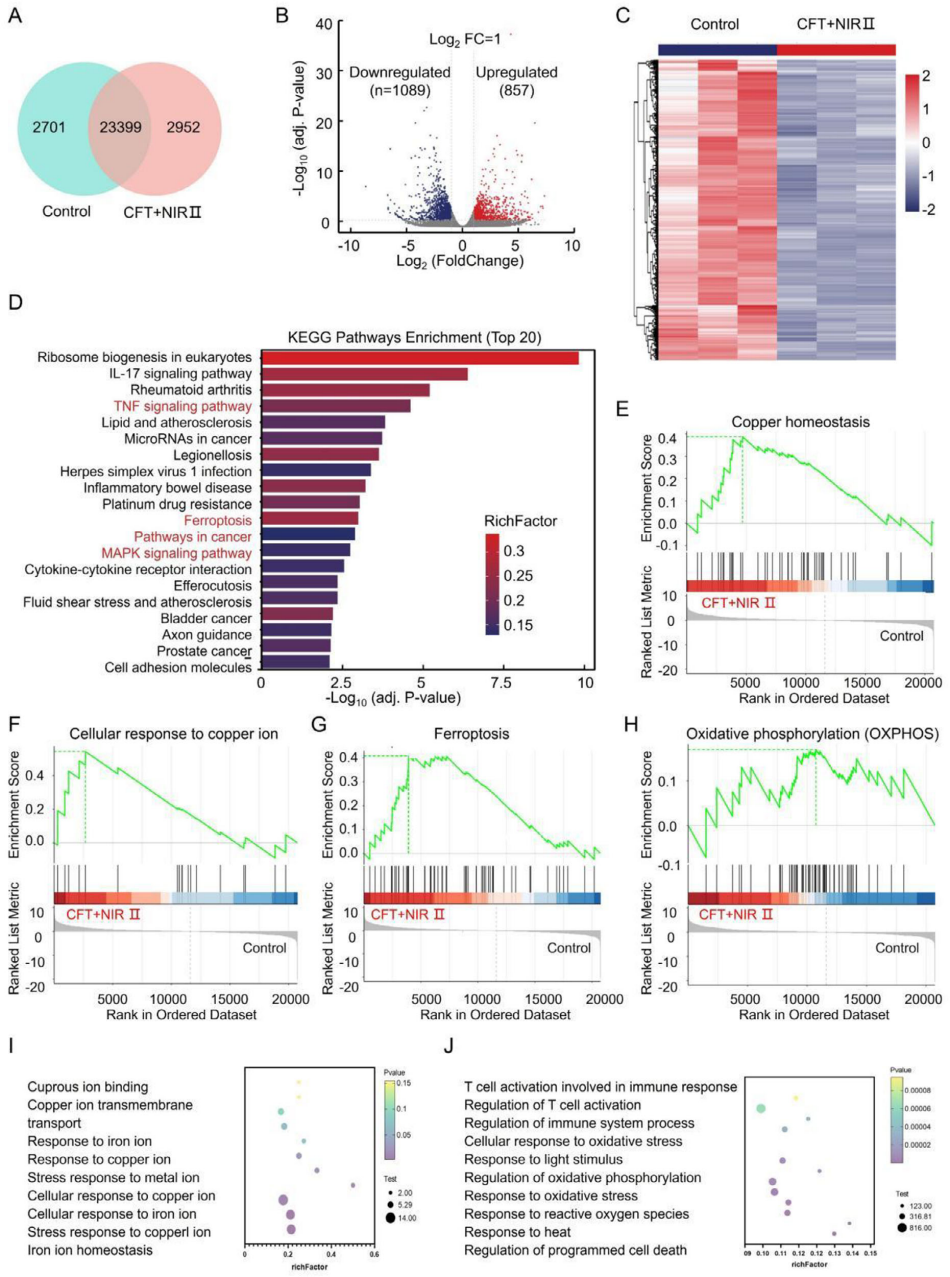
Anticaner biological mechanism of CFT. A) Venn diagram of the expressed genes, B) Volcano plot, C) heatmap of DEGs, D) KEGG enrichment analysis of DEGs, E–H) GSEA enrichment analysis of DEGs and I, J) KEGG enrichment analysis of differentially expressed ions and immune‐related pathways in CFT + NIR II and control groups (n = 3).

To further elucidate the biological mechanisms underlying CFT‐mediated therapy, RNA was extracted from 4T1 cells treated with CFT + NIR II, as well as from control groups, for transcriptomic analysis. As shown in **Figure** **6**A, a total of 23399 genes were co‐expressed in both the control and CFT + NIR II groups, while 2952 genes were exclusively expressed in the CFT + NIR II group. These findings suggest that CFT + NIR II treatment exerts a substantial impact on gene expression in 4T1 cells. Moreover, differential expression analysis using a volcano plot and heatmap identified 1946 genes that were significantly altered in response to CFT + NIR II treatment compared to the control, comprising 857 upregulated and 1089 downregulated genes (Figure 6B,C). To further investigate the functional relevance of these differentially expressed genes, enrichment analyses were performed. Kyoto Encyclopedia of Genes and Genomes (KEGG) pathway analysis (Figure 6D) revealed significant activation of key pathways in the CFT + NIR II group, including the mitogen‐activated protein kinase (MAPK) signaling pathway, the tumor necrosis factor (TNF) signaling pathway, and the ferroptosis pathway, providing insights into the molecular mechanisms driving the therapeutic effects of CFT + NIR II.

Oxidative stress induced by reactive oxygen species (ROS) is well known to cause irreversible damage to cellular components, including DNA, proteins, and lipids, ultimately leading to cell death. Previous studies have suggested that ROS‐mediated cytotoxicity is primarily driven by the MAPK and TNF signalling pathways.^[^ ^50^, ^51^ ^]^ Notably, compared to the control group, differentially expressed genes (DEGs) in the CFT + NIR II group were closely linked to these pathways (Figure 6D). Gene set enrichment analysis (GSEA) further revealed that gene expression changes in the CFT + NIR II group were positively correlated with copper homeostasis (Figure 6E), cellular responses to copper ions (Figure 6F), ferroptosis signaling (Figure 6G), and mitochondrial‐related pathways, including oxidative phosphorylation (OXPHOS) (Figure 6H) and the TCA cycle (Figure S20, Supporting Information). Additionally, KEGG pathway analysis highlighted the activation of cuproptosis and ferroptosis pathways (Figure 6I), OXPHOS pathways, and immune‐related pathways following CFT + NIR II treatment (Figure 6J), all of which are closely associated with ferroptosis‐ and cuproptosis‐induced mitochondrial dysfunction. Collectively, these findings demonstrate that CFT + NIR II treatment effectively suppresses cancer cells by inducing oxidative stress damage, mitochondrial dysfunction, and inflammatory responses, offering deeper insights into the intricate gene expression changes and signaling pathway alterations underlying its therapeutic effects.

### 2.6 In Vivo Therapeutic Performance of CFT

**Figure 7:**
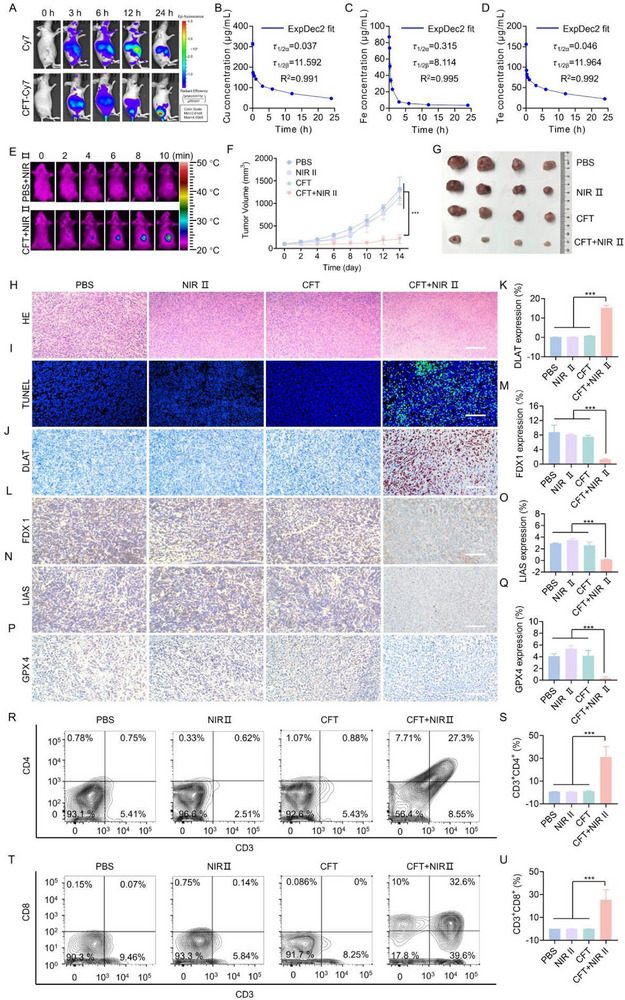
In vivo therapeutic performance of CFT in 4T1 tumor‐bearing mice. A) Fluorescence images of mice at 0, 3, 6, 12, and 24 h after intravenous injection of CFT‐Cy7 or free Cy7. B–D) The blood circulation curve of Cu (B), Fe (C) and Te (D). E) Infrared thermal images of 4T1 tumor‐bearing mice at 24 h post‐injection of PBS or CFT with 1064 nm laser radiation (1 W cm^−^^2^, 10 min). F) Tumor growth curves for different treatment groups over 14 days following intravenous injection (n = 4). G) Representative photographs of tumors from different groups at the end of treatment (n = 4). H, I) H&E and TUNEL staining images of tumor tissues from each group. IHC staining images of J) DLAT, L) FDX1, N) LIAS, and P) GPX4 in tumor sections obtained at the end of treatment and quantitative analyses of K) DLAT, M) FDX1, O) LIAS, and Q) GPX4. Typical flow cytometry plots of CD3^+^CD4^+^ T cells and CD3^+^CD8^+^ T cells in tumors after different treatments (R, T) and quantitative analysis of (S, U). Data are expressed as means ± s.d. (n = 4). Scale bar: 50 µm. Statistical significance was determined using Student's t‐test. *** *p* < 0.001.

The promising in vitro therapeutic outcomes of CFT prompted us to investigate its in vivo synergistic therapeutic effects in a BALB/c tumor‐bearing mouse model (n = 4). Initially, the tumor‐targeting capability of CFT was evaluated. We first investigated the tumor‐targeting capability of CFT by labeling it with Cy7 for non‐invasive fluorescence tracking. Following intravenous administration of CFT‐Cy7, whole‐body fluorescence imaging was performed at 0, 3, 6, 12, and 24 h post‐injection. In mice injected with free Cy7, the fluorescence signal was predominantly observed in the liver at early time points and rapidly diminished over time, with negligible tumor accumulation. In contrast, mice treated with CFT‐Cy7 displayed progressively intensified fluorescence at the tumor site over 24 h, indicative of efficient tumor enrichment, likely mediated by the enhanced permeability and retention (EPR) effect (**Figure** **7**A). Ex vivo fluorescence images of excised tumor and various organs at 24 h post‐injection (Figure S21, Supporting Information), together with ICP‐MS quantification of Cu, Fe, and Te elements (Figure S22A—C, Supporting Information), corroborated significant CFT retention within tumors, although notable accumulation was also detected in the liver and spleen due to sequestration by the reticuloendothelial system (RES). Pharmacokinetic profiling based on ICP‐MS quantification of blood samples collected at multiple time points (2 min, 8 min, 15 min, 30 min, 1 h, 2 h, 4 h, 8 h, and 24 h) yielded distribution half‐lives (τ_1/2_ (α)) of 0.037 h (Cu), 0.315 h (Fe), and 0.046 h (Te), and elimination half‐lives (τ_1/2_ (β)) of 11.592 h (Cu), 8.114 h (Fe), and 11.964 h (Te), respectively (Figure 7B–D). These β‐phase half‐lives (8–12 h) provide a favorable balance between prolonged systemic circulation—allowing sufficient tumor targeting via the EPR effect—and gradual clearance, thereby reducing the risk of long‐term tissue retention. ICP‐MS analysis of urine and feces at 3, 6, 12, 24, and 48 hours post‐injection indicated that the majority of injected CFT were excreted within 48 h (Figure S23A—C, Supporting Information). These findings are consistent with previous reports on Cu_2‐x_Te nanosheets, which also showed tumor accumulation through the EPR effect and clearance of Te‐containing species within a comparable timeframe.^[^ ^33^ ^]^ Although the exact half‐lives differ due to variations in material composition, surface functionalization, and dosage, both studies support that Te‐containing nanosheets can be efficiently cleared, minimizing the potential risk of toxic by‐products during degradation.

Next, the therapeutic efficacy of CFT in vivo was evaluated. The experimental design is depicted in Figure S24 (Supporting Information), where mice were divided into four groups: (1) PBS, (2) NIR II, (3) CFT, (4) CFT+NIR II. Mice in groups (2) and (4) were exposed to 1064 nm laser irradiation (1 W cm^−2^) for 10 min at 24 h post‐injection. The temperature of the tumor site was monitored by an infrared camera. Infrared thermal imaging (Figure 7E) revealed no significant temperature change in the control group following NIR II light exposure. In contrast, the CFT group exhibited a rapid temperature increase, from 26 °C to approximately 43 °C, following intravenous administration and subsequent NIR II laser irradiation. This result suggests that CFT effectively accumulated in the tumour region and induced mild localized hyperthermia under NIR II irradiation, which is promising for promoting cuproptosis and ferroptosis in the tumour. The antitumor effect of CFT was assessed over a 14‐day treatment period by measuring tumor volume every two days. Tumor growth curves (Figure 7F) and representative images of tumor tissues at the end of the treatment (Figure 7G) demonstrated a significant tumor regression in the CFT+NIR II group, in contrast to minimal inhibition observed in the other groups. The antitumor efficacy of CFT was further validated through hematoxylin and eosin (H&E) and TdT‐mediated dUTP nick‐end labeling (TUNEL) assays. H&E staining revealed marked tumor ablation in the CFT+NIR II group (Figure 7H). TUNEL staining showed strong green fluorescence signals (Figure 7I; Figure S25, Supporting Information), indicating pronounced apoptosis in the CFT+NIR II. In contrast, no significant tumor damage was observed in groups without NIR II exposure. Immunohistochemistry (IHC) staining demonstrated that the CFT+NIR II group exhibited the highest expression of DLAT (Figure 7J, K) and the lowest expression levels of FDX1, LIAS, and GPX4 (Figure 7L–Q). Immunofluorescence (IF) staining cooperated the above results, suggesting the induction of both cuproptosis and ferroptosis (Figure S26, Supporting Information). IHC staining also showed enhanced CRT expression (Figure S27A, C, Supporting Information) and reduced retention of HMGB1 (Figure S27B, D, Supporting Information) in the CFT+NIR II groups, indicating that CFT+NIR II treatment effectively triggered an immune response. Flow cytometry analysis of tumor‐infiltrating immune cells revealed significant increases in CD8^+^ cytotoxic T cells and CD4^+^ helper T cells in the CFT+NIR‐II group compared to controls (Figure 7R–U), further confirming activation of both innate and adaptive immune compartments. IF staining for CD3 and CD8 (Figure S28, Supporting Information) corroborated these findings, showing markedly higher infiltration of cytotoxic T lymphocytes. These data collectively indicate that CFT+NIR‐II treatment induces ICD and stimulates T‐cell–mediated immunity, thereby contributing to a potent anti‐tumor effect with pronounced abscopal responses, likely driven by combined cuproptosis and ferroptosis mechanisms. Throughout the treatment period, the body weight of mice in all groups showed a steady increase (Figure S29, Supporting Information), and no significant abnormalities or organ damage were observed in the heart, liver, spleen, lungs, or kidneys (Figure S30, Supporting Information), indicating the excellent biocompatibility of CFT.

## 3 Conclusion

In summary, we have developed a synergistic therapeutic strategy that combines internal TME‐activated and external NIR II irradiation to promote cuproptosis and ferroptosis for the effective inhibition of TNBC. Under the mildly acidic conditions of the TME, the biodegradable CFT nanosheets released Fe^2+^ and Cu^2+^, initiating cyclic Fenton reactions and depleting endogenous GSH, thereby amplifying oxidative stress. The mild local hyperthermia induced by CFT further accelerated these catalytic reactions, enhancing ROS production and GSH consumption under NIR II irradiation, which intensified oxidative stress. Accumulated Cu^+^ ions induced DLAT aggregation and Fe–S cluster proteins, triggering cuproptosis. Simultaneously, the resultant oxidative stress, coupled with abundant iron ions, promoted LPO and inactivated GPX4, thereby driving ferroptosis. This dual induction of cuproptosis and ferroptosis reduced Cu^+^ efflux, released ATP, and ultimately triggered a potent immune response. Integrated transcriptomic analyses revealed that the combined effects of cuproptosis and ferroptosis induced mitochondrial dysfunction through the TCA cycle, respiratory electron transport, OXPHOS pathways, and copper homeostasis, alongside multiple immune‐related pathways. Collectively, this integrated therapeutic strategy, combining cuproptosis and ferroptosis, presents a promising new approach for the clinical treatment of TNBC.

## 4 Experimental Section

### Materials

CuFeTe_2_ powder was obtained from Nanjing Muke Nanotechnology corporation. Thiol‐terminated PEG (PEG‐SH, Mw = 5000) was supplied from Ponsure. DMEM medium, and phosphate buffer saline (PBS) were all acquired from Procell. CCK‐8 was purchased from APExBIO. MitoSox Red, Hoechst 33342, was obtained from Beyotime (Shanghai, China). FerroOrange was obtained from Dojindo Laboratories (Japan). Lpd peroxide probe and C11‐BODIPY^581/591^ was obtained from MedChemExpress (MCE) (America). BioTracker Green Copper Live Cell Dye was obtained from Merck KGAA (Japan). Distilled water for laboratory was obtained from the Milli‐Q System.

### Characterization

The morphology and elemental distribution were characterized by TEM (FEI Tecnai G20). XRD pattern was recorded by Rigaku‐Dmax 2500 diffractometer. X‐ray photoelectron spectroscopy (XPS) spectra was acquired from ESCALAB 250Xi. UV‐Vis‐NIR spectra were recorded with UH5700 UV‐Vis/visible/NIR spectrometer. Zeta potential and DLS were measured by Malvern Nanosizer ZS. Fluorescence images were captured using fluorescence microscope (Nikon Ti‐S) or the confocal laser scanning microscope (CLSM, Leica TCS SP8). ICP‐MS analysis were performed by Thermo Fisher iCAP RQ. ESR spectra were recorded by a Bruker A300 spectrometer. TG analysis was performed on TA Q600. The thickness of CFT was characterized using AFM (bruker dimension icon).

### Preparation of CFT

40 mg of bulk CuFeTe_2_ nanosheet were added into 20 mL of pyrrolidone at room temperature and sonicated at low temperature for 36 hours until the particles were completely dissolved, resulting in CuFeTe_2_‐pyrrolidone. Then, 30 mg of CuFeTe_2_‐pyrrolidone, 60 mg of PVP k30, and 60 mL of ethanol were mixed and heated at 50 °C under reflux for 24 hours to obtain CFT‐PVP. After washing with deionized water, freshly prepared SH‐PEG was added dropwise, allowing the thiol groups to coordinate to the surface via strong thiol–metal interactions. The mixture was incubated overnight in the dark with stirring, followed by centrifugation (7000 rpm, 15 min) and washed with deionized water to remove free SH‐PEG from the supernatant. Finally, the purified CFT was lyophilized, yielding approximately 53% of the total precursor mass.

### Preparation of CFT‐Cy7

CFT was first functionalized by SH‐PEG‐NH_2_ for further binding to the carboxyl groups of Cy‐7. In detail, 20 mL of CFT‐PVP aqueous solution was ultrasonicated for 1 h and SH‐PEG‐NH_2_ aqueous solution (500 µg mL^−1^) was added dropwise. After overnight stirring under dark condition, the above suspension was centrifuged at 7000 rpm, washed with deionized water for several times and dispersed in 20 mL pure water, named CFT‐NH_2_. At the same time, 1 mL of EDC (2.4 mg mL^−1^) and 1 mL of NHS (2 mg mL^−1^) were infused with 1 mL of Cy‐7 aqueous solution (1 mg mL^−1^) at stirring for 24 h under dark condition. After concentrated and washed with deionized water for three times, the above CFT‐NH_2_ aqueous solution was added and stirred for 24 h. The yield Cy‐7 labeled CFT were collected by centrifuged and washed with water three times.

### Biodegradability of CFT

CFT (1 mg) were dispersed in 5 mL of PBS solution with different pH values (5.5, 6.5, and 7.4) and then shaken at 200 r min^−1^ at room temperature. At 0 h, 24 h, and 48 h, the resulting solution was centrifuged at 12,000 rpm for 5 min. The supernatant was obtained for ICP‐MS analysis while the precipitate was used to observe the morphology using TEM.

### ROS‐Generating Activity of CFT

The ROS‐generating capability of CFT was elevated using the TMB chromogenic assay in the presence of H_2_O_2_. Briefly, TMB and H_2_O_2_ solutions were added into CFT suspensions at different pH values (5.5, 6.5, and 7.4) and incubated for 30 min. The absorbance at 652 nm was recorded spectrophotometrically. The final concentration of CFT was 100 µg mL^−1^, H_2_O_2_ was 40 mM, and DMPO was 50 mM, respectively. Samples without H_2_O_2_ served as negative controls. The concentration‐dependent POD‐like activity was assessed at pH 5.5 by varying CFT concentrations (0, 12, 25, 50, and 100 µg mL^−1^). The mild hyperthermia‐enhanced ROS‐generating activity of CFT were conducted by the same method above and irradiated the mixture with a 1064 nm laser (1 W cm^−2^, 10 min) and incubated for another 20 min.

·OH generation was further verified by ESR spectroscopy. In brief, CFT, H_2_O_2_, and DMPO were mixed in capillary tubes at the same final concentrations described above, and ESR spectra were recorded. Samples without H_2_O_2_ served as negative controls. For hyperthermia‐enhanced ESR measurements, the mixture was irradiated with a 1064 nm laser (1 W cm^−2^, 10 min) and immediately subjected to ESR analysis.

### GSH Depletion of CFT

CFT dispersions at different pH values (5.5, 6.5, and 7.4) were incubated with GSH (5 mM) at room temperature. Subsequently, DTNB, 0.2 mM) was added, and the absorbance at 412 nm was monitored to quantify the residual GSH. The final concentration of CFT was 100 µg mL^−1^. To assess the effect of mild hyperthermia, the same protocol was performed with additional irradiation by a 1064 nm laser (1 W cm^−2^, 10 min). PBS at corresponding pH values (5.5, 6.5, and 7.4) served as the control.

### Photothermal Performance Test of CFT

The photothermal properties of CFT were evaluated by dispersing CFT at various concentrations (0, 50, 100, and 200 µg mL^−1^) in a quartz cuvette and irradiated with 1064 nm laser (1 W cm^−2^, 10 min). The temperature profile was recorded every 30 s. Infrared thermography (Fotric 1204) was used to capture infrared thermal images to record the temperature variations of CFT solutions (100 µg mL^−1^, 1 W cm^−2^, 10 min). To evaluate the photothermal conversion, the laser was turned off after 10 min of irradiation and the temperature was allowed to cool for 10 min. The photothermal conversion efficiency (η) was calculated according to Roper's report by Equation (1), where T_max_ was the maximum temperature of CFT solutions; T_max, water_ was that of pure water; *I* was the laser power; A_1064_ was the absorption of CFT solutions dispersed in water at 1064 nm. The value of hS can be obtained through Equation (2), where m_i_ and C_p, i_ denoting the mass and heat capacity, respectively. The system time constant (τ_s_) was derived from the linear fitting of the cooling data using Equation (3).

(1) $$\eta =\frac{hS\left(T_{\max}-T_{\max,water}\right)}{I\left(1-10^{-A_{1064}}\right)}$$

(2) $$hS=\frac{\sum_{i}m_{i}C_{p,i}}{\tau_{s}}$$

(3) $$\tau_{s}=-\ln\left(\frac{T\left(t\right)-T_{sur}}{T_{\max}-T_{sur}}\right)$$

In this study, m = 1 g, C_water_ = 4.2 J/(g/°C)), τ_s_ = 491.6 s and hS = 0.0085 W/°C. With (T_Max_‐T_Surr_) = 18 °C, I_1064_ = 1 W, and A_1064_ = 0.216, the η of CFT under 1064 nm irradiation was calculated to be 39.2%.

Photothermal stability was further assessed by irradiating CFT (100 µg mL^−1^) at different pH values (5.5, 6.5, and 7.4) with a 1064 nm laser (1 W cm^−2^, 10 min) for four consecutive heating–cooling cycles.

### Cell Culture

NIH‐3T3 (mouse embryonic fibroblast, Mus musculus, male, RRID:CVCL_0594), HEK 293T (human embryonic kidney, female fetus, RRID:CVCL_0063), RAW 264.7 (murine monocyte/macrophage, Mus musculus, male, RRID:CVCL_0493), 4T1 (murine mammary carcinoma, Mus musculus, female, RRID:CVCL_0125), MDA‐MB‐231 (human breast adenocarcinoma, Homo sapiens, female, RRID:CVCL_0062), and B16‐F10 (murine melanoma, Mus musculus, male, RRID:CVCL_0159) were used in this study. All cell lines were obtained from the Cell Bank of the Chinese Academy of Sciences (Shanghai, China) between 2020 and 2024. The identity of each cell line was authenticated by short tandem repeat (STR) profiling provided by the supplier, showing >90% match with the reference profiles, and all were confirmed not to be listed as misidentified in the International Cell Line Authentication Committee (ICLAC) or Cellosaurus databases. All cell lines were routinely tested for mycoplasma contamination prior to experiments and confirmed negative. Cells were used within 3–20 passages after thawing to minimize genetic drift.

NIH‐3T3 cells were cultured in DMEM supplemented with 10% bovine calf serum and 1% penicillin/streptomycin. HEK 293T, RAW 264.7, 4T1 and MDA‐MB‐231 cells were cultured in DMEM containing 10% FBS and 1% penicillin/streptomycin. B16‐F10 cells were cultured in Roswell Park Memorial Institute 1640 (RPMI 1640) containing 10% FBS and 1% penicillin/streptomycin. Cells were maintained at 37 °C in a humidified incubator with 5% CO_2_, the medium was refreshed every three days, and cells were harvested by trypsinization prior to plating.

### In Vitro Cytotoxicity Evaluation

NIH‐3T3, HEK 293T, RAW 264.7, 4T1, MDA‐MB‐231 and B16‐F10 cells (10^4^ cells well^−1^) were seeded in 96‐well plates for 12 h. Then, 100 µL of CFT solutions at various concentrations (0, 6, 12, 25, 50, and 100 µg mL^−1^) at different pH (7.4, 6.5) were added. After 24 h of incubation, cell viability was assessed using a CCK‐8 assay according to the manufacturer's protocol.

### Synergistic Phototherapeutic Assessment

To evaluate the combined phototherapeutic effect of CFT under NIR‐II irradiation, 4T1 cells were incubated with CFT for 6 h. The cells were then irradiated with a 1064 nm laser (1 W cm^−2^, 10 min) and further incubated for 18 h. Cell viability was then assessed using a CCK‐8 assay according to the manufacturer's protocol.

For live/dead cell staining, cells were washed with PBS and incubated with calcein AM (2 µM) and PI (4 µM) for 1.5 h in the dark. After washing with PBS, fluorescence images were acquired using a fluorescence microscope to evaluate cell viability.

### Intracellular ROS Detection

Intracellular ROS generation was detected using DCFH‐DA (ROS Assay Kit) as the fluorescent probe. 4T1 cells (8×10^4^ cells/well) were seeded in 12‐well plates and cultured for 12 h. Thereafter, 800 µL of CFT solutions at different concentrations (0, 6, 12, 25, 50, and 100 µg mL^−1^) were added. After 6 h of incubation, the cells were irradiated with a 1064 nm laser (1 W cm^−2^, 10 min) and cultured for an additional 18 h. Control groups were incubated in the dark for 24 h. The cells were then stained with DCFH‐DA (20 µM) for 2 h, and intracellular fluorescence was analyzed by both fluorescence microscopy and flow cytometry.

### Western blot Analysis of Protein Expression

4T1 cells (1.6×10^5^ cells/well) were seeded in 6‐well plates and cultured for 12 h. Cells were then treated with 1.6 mL of CFT (100 µg mL^−1^) for 6 h, followed by 1064 nm laser irradiation (1 W cm^−2^, 10 min) and an additional 18 h incubation. Untreated, CFT‐only, and NIR‐II‐only groups served as controls. The cells were washed twice with PBS, lysed with cell lysis buffer, and centrifugated to quantify the protein content to 20 µg. Then, the proteins were separated by SDS–polyacrylamide gel electrophoresis (PAGE) gel electrophoresis and transferred onto PVDF membrane. Membranes were blocked with 5% skim milk for 2 h, followed by overnight incubation with primary antibodies at 4 °C. After washing with TBST, membranes were incubated with horseradish peroxidase (HRP)‐conjugated secondary antibodies for 1 h at room temperature. Protein bands were visualized using a Tanon 5200 Multi‐Imaging System (Tanon Science & Technology).

### Characterization of Mitochondrial Damage

4T1 cells (1.6×10^5^ cells/well) were seeded in 6‐well plates and cultured for 12 h. Cells were then treated with 1.6 mL of CFT (100 µg mL^−1^) for 6 h, followed by 1064 nm laser irradiation (1 W cm^−2^, 10 min) and an additional 18 h incubation. Untreated, CFT‐only, and NIR‐II‐only groups served as controls. Cells were then stained with Hoechst 33342 (10 µg mL^−1^), MitoSox (5 µM) or JC‐1 (10 µg mL^−1^) for 15 min and imaged by fluorescence microscope.

### Bio‐TEM Morphology Observation

4T1 cells (4×10^4^ cells/well) were seeded in 24‐well plates and cultured for 12 h. Cells were then treated with 1.6 mL of CFT (100 µg mL^−1^) for 6 h, followed by 1064 nm laser irradiation (1 W cm^−2^, 10 min) and an additional 18 h incubation. Untreated, CFT‐only, and NIR‐II‐only groups served as controls. Cells were fixed with 3% glutaraldehyde, dehydrated with alcohol and embedded in epoxy resin. Ultrathin sections were cut and mitochondrial morphology in the different samples was observed via TEM.

### Intracellular Cu^+^ Detection

4T1 cells (8×10^4^ cells/well) were seeded in 12‐well plates and cultured for 12 h. Cells were then treated with 1.6 mL of CFT (100 µg mL^−1^) for 6 h, followed by 1064 nm laser irradiation (1 W cm^−2^, 10 min) and an additional 18 h incubation. Untreated, CFT‐only, and NIR‐II‐only groups served as controls. After staining with Hoechst 33342 and BioTracker Green Copper Live Cell Dye, the cells were imaged by CLSM.

### Intracellular Fe^2+^ Detection

4T1 cells were treated as described above, stained with Hoechst 33342 and FerroOrange and imaged by fluorescence microscope.

### Cellular LPO Detection

4T1 cells were treated as described above and stained with Hoechst 33342 and C11‐BODIPY ^581/591^. CLSM was used to evaluate lipid peroxidation.

### Intracellular GSH Detection

Intracellular GSH levels were quantified by DTNB assay. After the same treatment protocol, cells were lysed, and the extracts were incubated with DTNB for 30 min. Absorbance at 412 nm was recorded.

### ATP Release Measurement

Following identical treatment conditions, ATP levels were measured using an ATP assay kit (Nanjing Jiancheng Bioengineering Institute, A095‐1‐1) according to the manufacturer's protocol.

### Tumor Xenograft Model

Female BALB/c mice (6 weeks old) were subcutaneously injected with 4T1 cells (1×10^7^ cells/well) into the dorsal flank to establish primary tumors. Tumor volumes were measured with calipers, and mice bearing tumors of ≈100 mm^3^ were randomized into four groups (n = 4 per group). All animal procedures were approved by the Animal Ethics Committee of Shanxi Medical University (SYDL2025016).

### In Vivo Biodistribution and Pharmacokinetics

In vivo biodistribution were analyzed by fluorescence imaging and ICP‐MS. 4T1 tumor‐bearing mice (tumor ≈100 mm^3^) were intravenously injected with CFT@Cy7 nanoformulations (20 mg kg^−1^, 200 µL). Fluorescence images of the mice were acquired at 0, 3, 6, 12, and 24 h post‐injection under gas anesthesia. At the 24 h time point, major organs and tumors were harvested for ex vivo fluorescence imaging.

For ICP‐MS analysis, mice injected with CFT (20 mg kg^−1^, 200 µL) were sacrificed at 3, 6, 12, 24, and 48 h, and major organs and tumors were analyzed. Blood samples were collected at 2, 8, 15, 30 min, 1, 2, 4, 8, and 24 h post‐injection for pharmacokinetic analysis. Excretion was assessed by collecting urine and feces at 3, 6, 12, 24, and 48 h in metabolic cages, followed by digestion in 70% HNO_3_ and quantification of Cu, Fe, and Te by ICP–MS.

### In Vivo Infrared Thermal Imaging

Tumor‐bearing mice (≈100 mm^3^) were intravenously injected with CFT (20 mg kg^−1^, 200 µL). After 24 h injection, tumor sites were irradiated with NIR II light (1064 nm, 1 W cm^−2^) for 10 min. Real‐time thermal imaging was recorded every 2 min using a Fotric 1204 infrared camera.

### In Vivo Therapeutic Effect of CFT

Tumor‐bearing mice (≈100 mm^3^) were divided into 4 groups (4 mice in each group): (1) PBS; (2) NIR II; (3) CFT; (4) CFT +NIR II. For group (2) and (4), the region of tumor were illuminated with 1064 nm laser. The tumor sizes and body weights of the mice in different groups were measured every other day. The tumor volume was calculated according to the formula: Width^2^ × length/2. At the end of treatment on the 14th day, the main organs, and tumors of mice in each group were harvested for H&E staining. Tumors were further used for TUNEL staining, GPX‐4, DLAT, LIAS, FDX‐1, HMGB‐1, CRT, CD3, and CD8 immunohistochemistry staining.

### Intratumoral T Cell Analysis by Flow Cytometry

Tumor‐bearing mice (≈100 mm^3^) were randomized into 4 groups (4 mice in each group): (1) PBS; (2) NIR II; (3) CFT; (4) CFT +NIR II. For group (2) and (4), the region of tumor were illuminated with 1064 nm laser. On day 14 post‐treatment, tumors were excised and enzymatically dissociated using a digestion buffer containing collagenase I (1 mg mL^−1^), collagenase IV (1 mg mL^−1^), and DNase I (0.2 mg mL^−1^) to obtain single‐cell suspensions. Cells were stained with fluorochrome‐conjugated antibodies against CD3, CD4, and CD8 for T cell subset analysis (FITC anti‐mouse CD3, PE anti‐mouse CD4, APC anti‐mouse CD8). Flow cytometry was performed to quantify CD3⁺CD4⁺ and CD3⁺CD8⁺ T cell populations.

### Statistical Analyses

Quantitative results were represented as the mean ± standard deviation (s.d.), indicated by error bars in all graphs. Statistical analyses were conducted using GraphPad Prism 10.1.2. Statistical significance among multiple groups was assessed using one‐way analysis of variance (ANOVA), while comparisons between two groups were conducted using Student's t‐test. *P* value of less than 0.05 was considered statistically significant (*p < 0.05, ** *p* < 0.01, *** *p* < 0.001.).

## Conflict of Interest

The authors declare no conflict of interest.

## Author Contributions

M.L., J.Z., M.Y. contributed equally to this work. M.L. carried out the synthesis, characterization of CFT, data analysis, and Figure preparation, Jian Zheng performed the biological assessments in vivo, M.Y. performed the biological assessments in vivo, Q.W. contributed to the data analysis. Y.Y., N.S., X.Y., and L.W. assisted with transcriptomic data analysis. T.S. assisted with the schematic illustration for the manuscript. A.L. and R.L. guided the animal experiments. J.C. contributed to funding acquisition and manuscript revision. X.L. performed materials characterization and assisted with manuscript editing. F.C. performed interpretation of results, manuscript revision, project administration and supervision. Y.F. performed conceptualization, original draft preparation, manuscript revision, project administration and supervision.

## Supporting information



Supporting Information

## Data Availability

The data that support the findings of this study are available from the corresponding author upon reasonable request.
