# Supplementary material for: Stimuli‐Responsive CuFeTe2 Nanosheets for Amplified Cuproptosis/Ferroptosis in Triple‐Negative Breast Cancer Therapy
Source: Adv Sci (Weinh). 2025 Oct 24;13(3):e05739. doi: 10.1002/advs.202505739 (PMC12806277; doi:10.1002/advs.202505739)
Supplement: Supplementary file 1 — Supporting Information [file ADVS-13-e05739-s001.docx]

Supporting Information

**Stimuli-responsive CuFeTe_2_ Nanosheets for Amplified Cuproptosis/Ferroptosis in Triple-Negative Breast Cancer Therapy**

*Molin Liu^#^, Jian Zheng^#^, Mengqi Yu^#^, Qirui Wang, Yi Yuan, Nannan Shao, Xiaoliang Yang, Tianxi Shen, Li Wang, Aiyun Li, Rui Liu, Jimin Cao*, Xi Liu*, Fangfang Cao*, Yanlin Feng^*^*

^#^ These authors contributed equally to this work.

^*^ Corresponding Authors.

Email: caojimin@sxmu.edu.cn, [liuxipla@163.com](mailto:liuxipla@163.com), [fangfangcao@buaa.edu.cn](mailto:fangfangcao@buaa.edu.cn), feng@sxmu.edu.cn

M. Liu, Y. Yuan, A. Li, R. Liu, J. Cao, Y. Feng

Department of Cardiology, the First Hospital of Shanxi Medical University, and Key Laboratory of Cellular Physiology at Shanxi Medical University, Ministry of Education, Taiyuan, 030001, China

J. Zheng

Shanxi Province Cancer Hospital, Chinese Academy of Medical Sciences, Cancer Hospital Affiliated to Shanxi Medical University, Taiyuan, 030001, China

M. Yu, Q. Wang, F. Cao

School of Engineering Medicine, Beihang University, Beijing, 100191, China.

N. Shao, X. Yang, L. Wang

CAS Key Laboratory for Nano-Bio Interface, Suzhou Institute of Nano-Tech and Nano-Bionics, Chinese Academy of Sciences, Suzhou 215123, China

1. Shen.

Nanchang People’s Hospital, Nanchang 330000, China.

X. Liu

Medical Innovation Research Division Chinese PLA General Hospital, Beijing, 100048, China


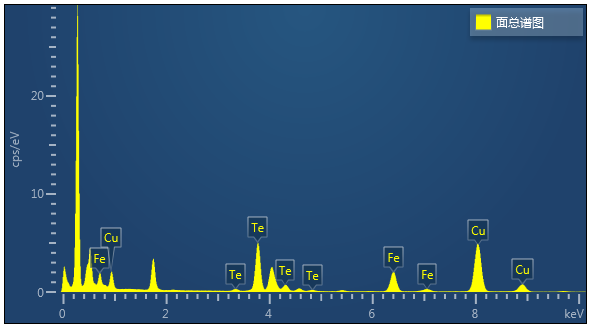


**Figure S1.** EDX spectrum of CFT.


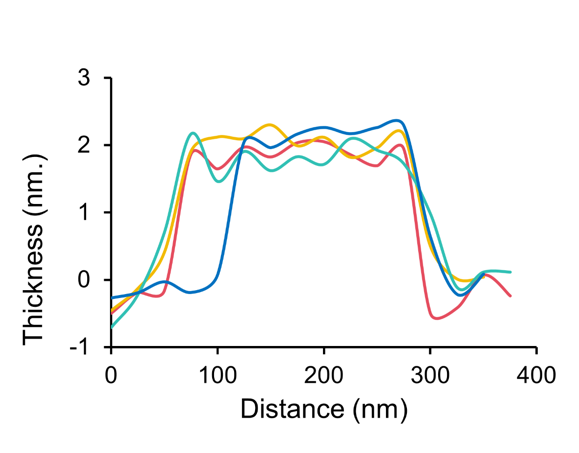


**Figure S2.** Corresponding height analysis of CFT in AFM images in Figure 2D.


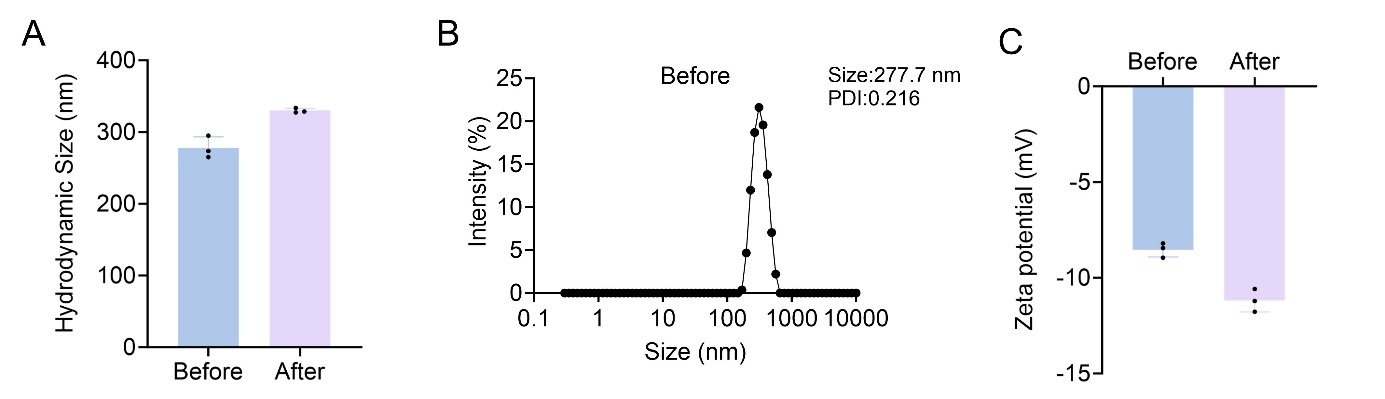


**Figure S3.** (A) Hydrodynamic size of CFT before and after PEGylation. (B) Size distribution of CFT before PEGylation in H_2_O. (C) Zeta potential of CFT before and after PEGylation.


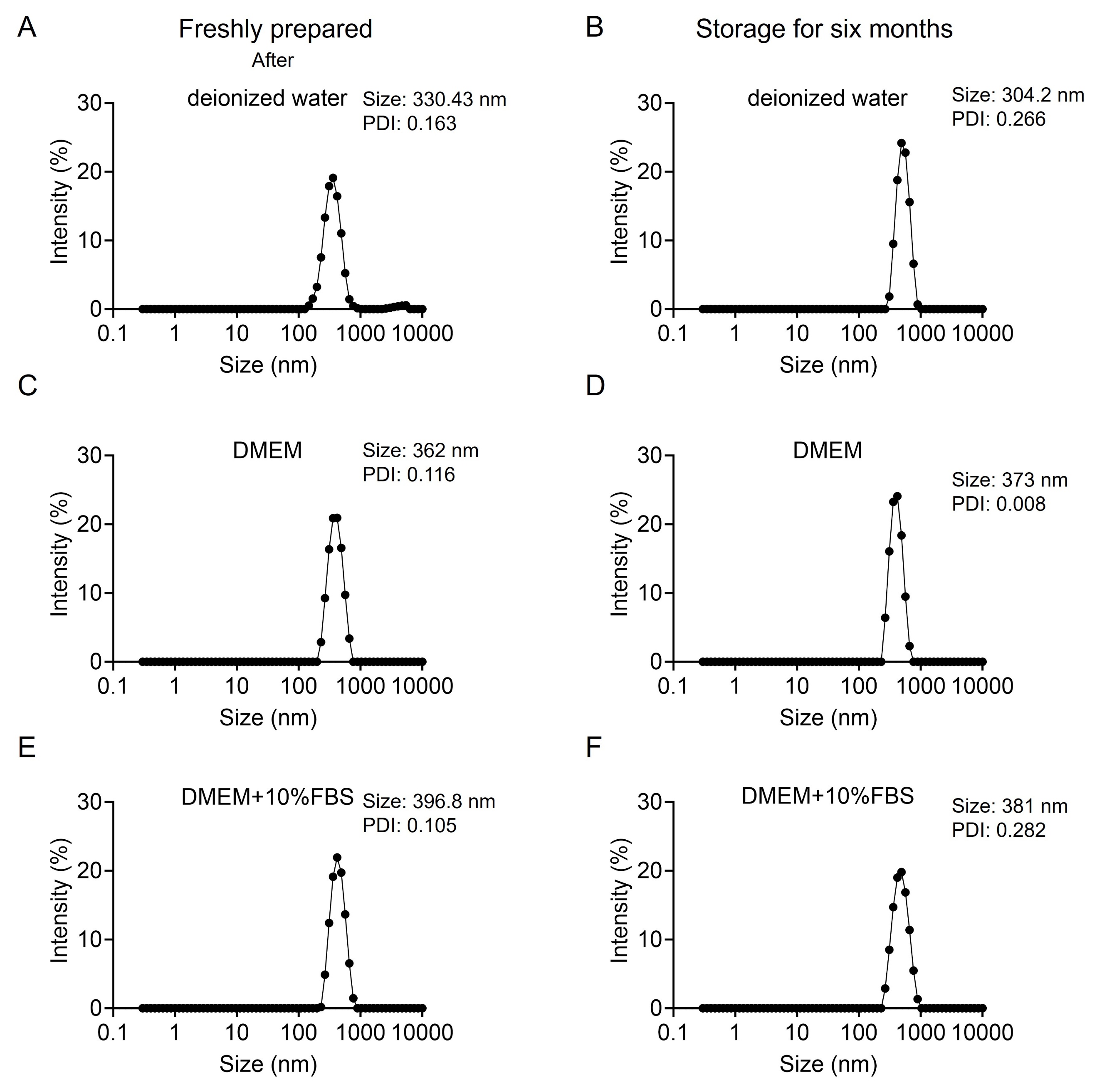


**Figure S4.** Size distribution of CFT after PEGylation in H_2_O (A, B), DMEM (C, D) and DMEM containing 10% FBS (E, F). A, C, E refered to freshly prepared CFT and B, D, F efered to DLS test after storage for 6 months.


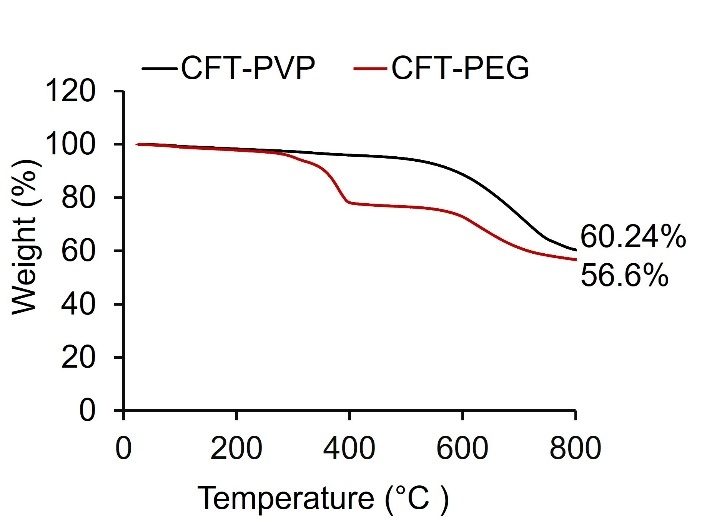


**Figure S5.** Thermogravimetric analysis of CFT-PVP (before PEGylation) and CFT-PEG (after PEGylation).


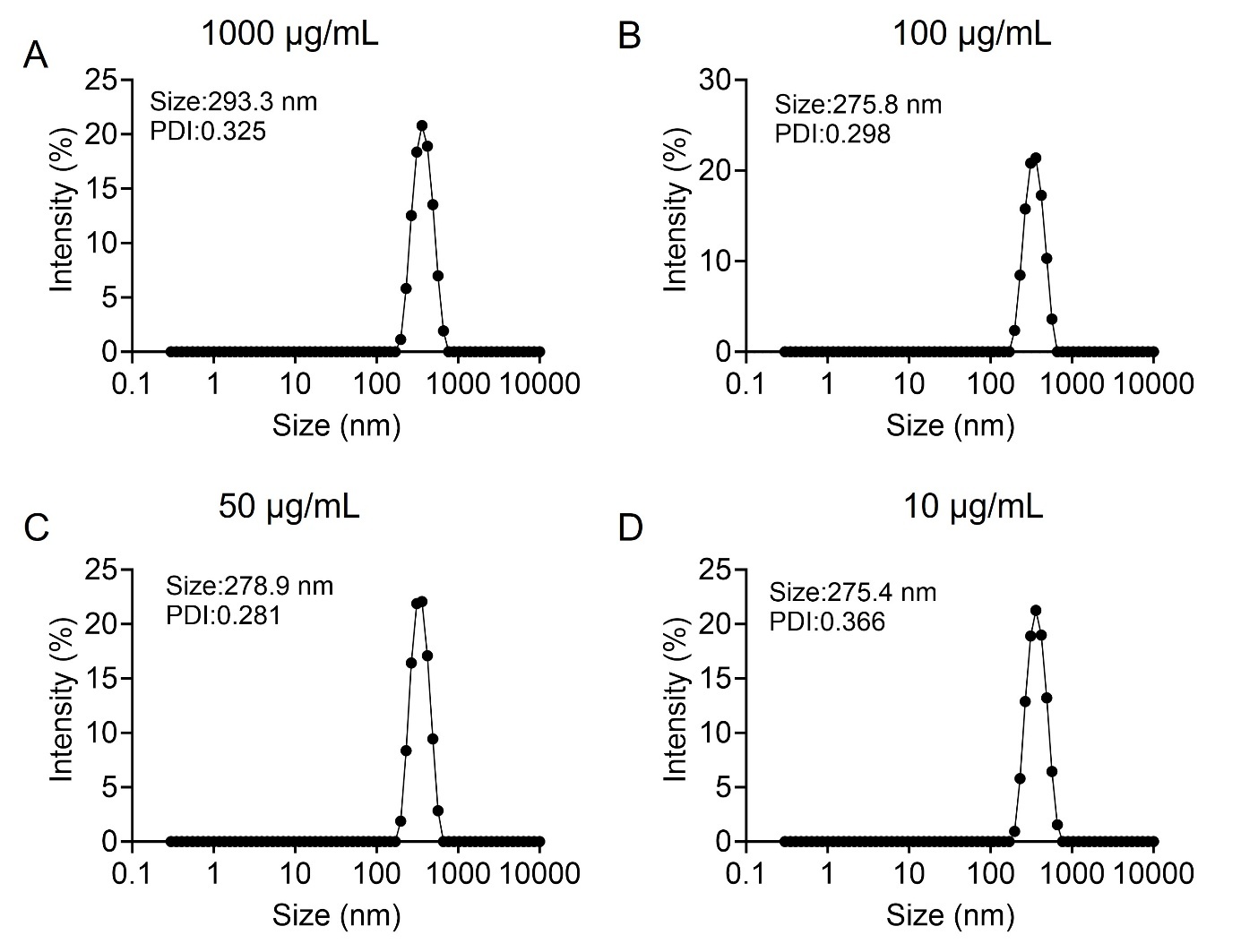


**Figure S6.** Size distribution of CFT of different concentrations.


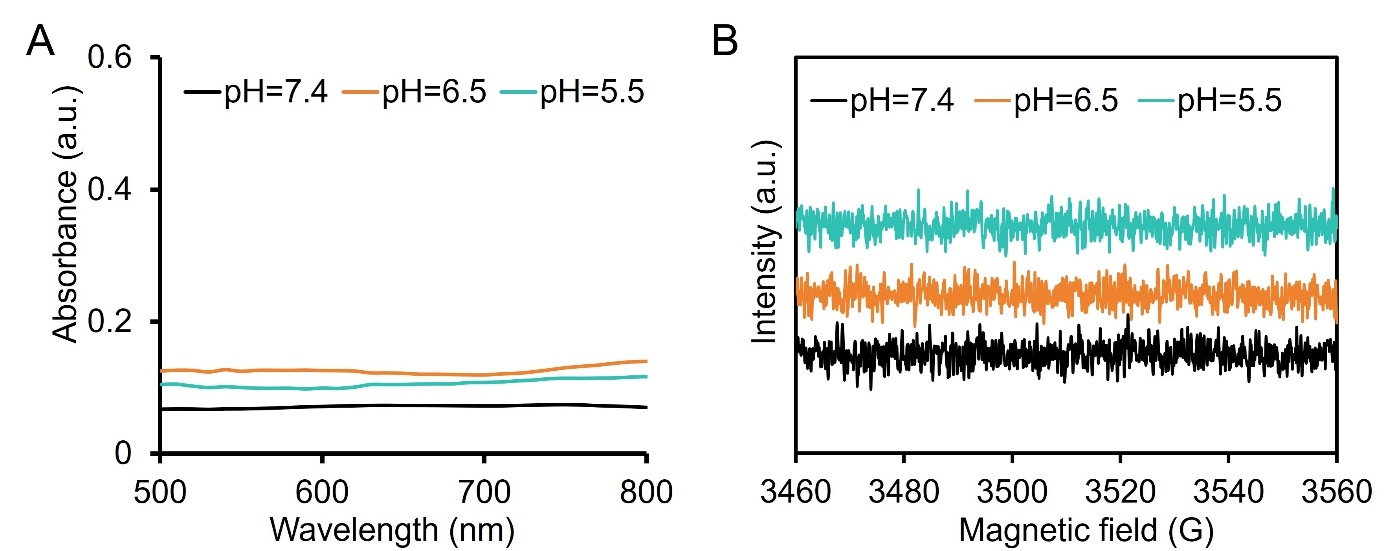


**Figure S7.** (A) UV-vis absorption spectra of oxidized TMB recorded under different pH conditions values in the presence CFT but without H_2_O_2_. (B) DMPO spin-trapping ESR spectra of CFT under different pH conditions values in the absence H_2_O_2_.


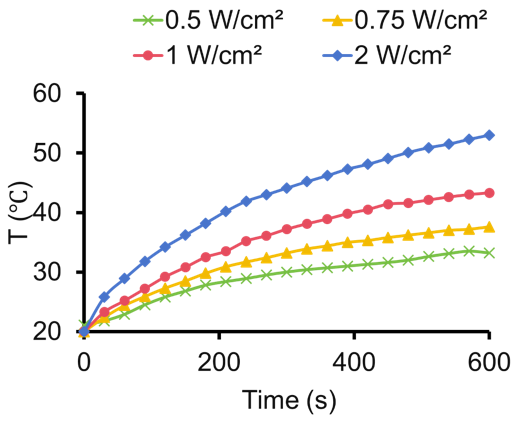


**Figure S8.** Temperature elevation of CFT (100 μg/mL) with 1064 nm laser irradiation with different power density.


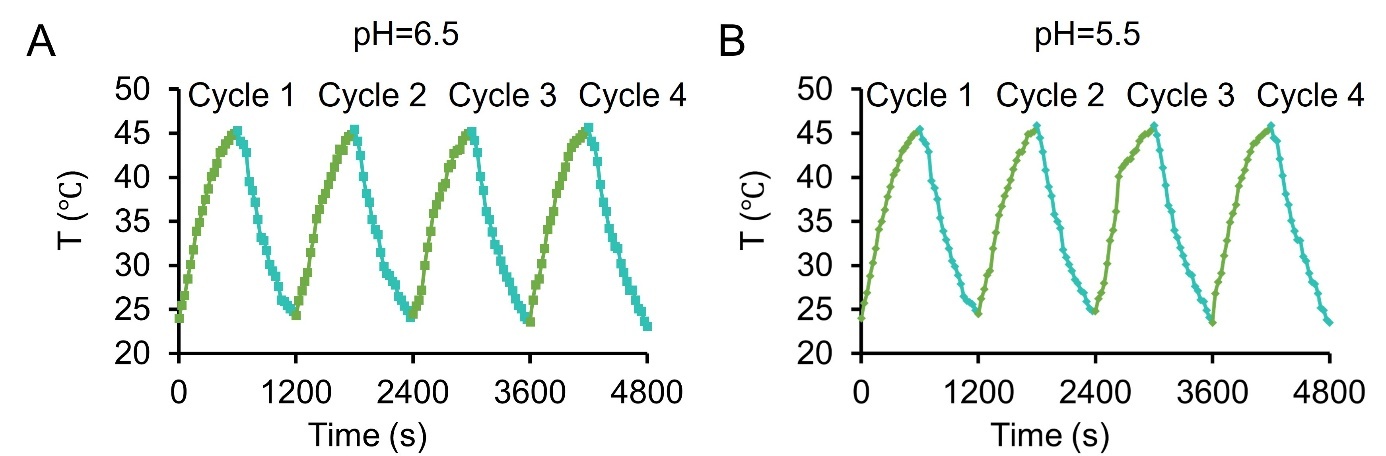


**Figure S9.** Photothermal heating and natural cooling cycles of CFT at pH=6.5 (A) and pH=5.5 (B).


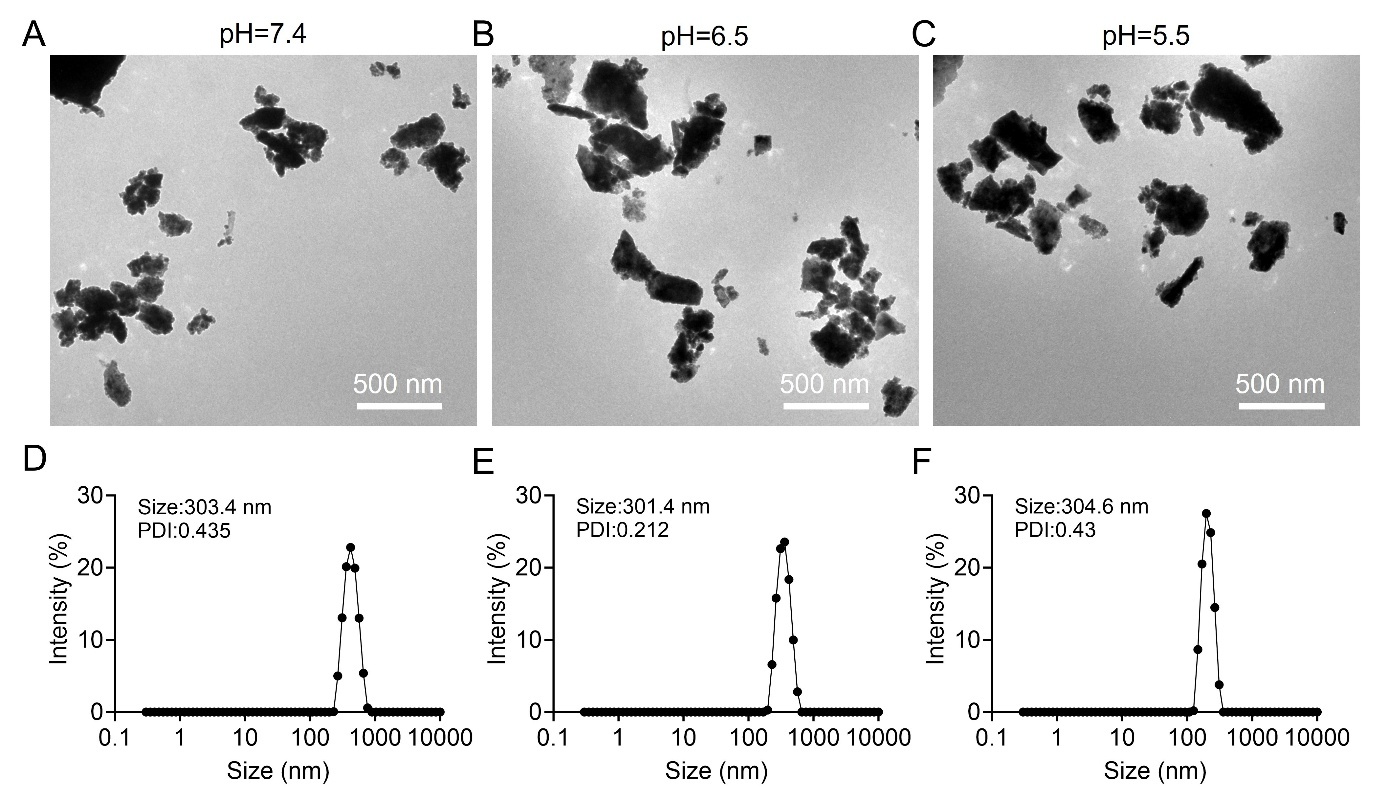


**Figure S10.** TEM images tested after 10 min of 1064 nm laser irradiation at pH=7.4 (A), pH=6.5 (B) and pH=5.5 (C) and corresponding size distributions after irradiation at pH=7.4 (D), pH=6.5 (E) and pH=5.5 (F).


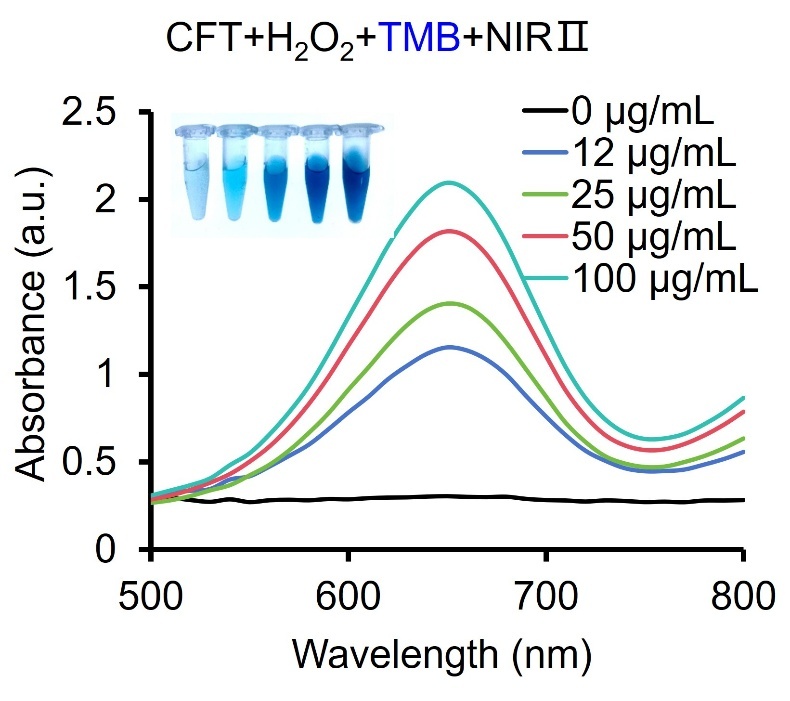


**Figure S11.** UV-vis absorption spectra of oxidized TMB recorded under different CFT concentrations with 1064 nm laser irradiation (1 W/cm^2^, 10 min).


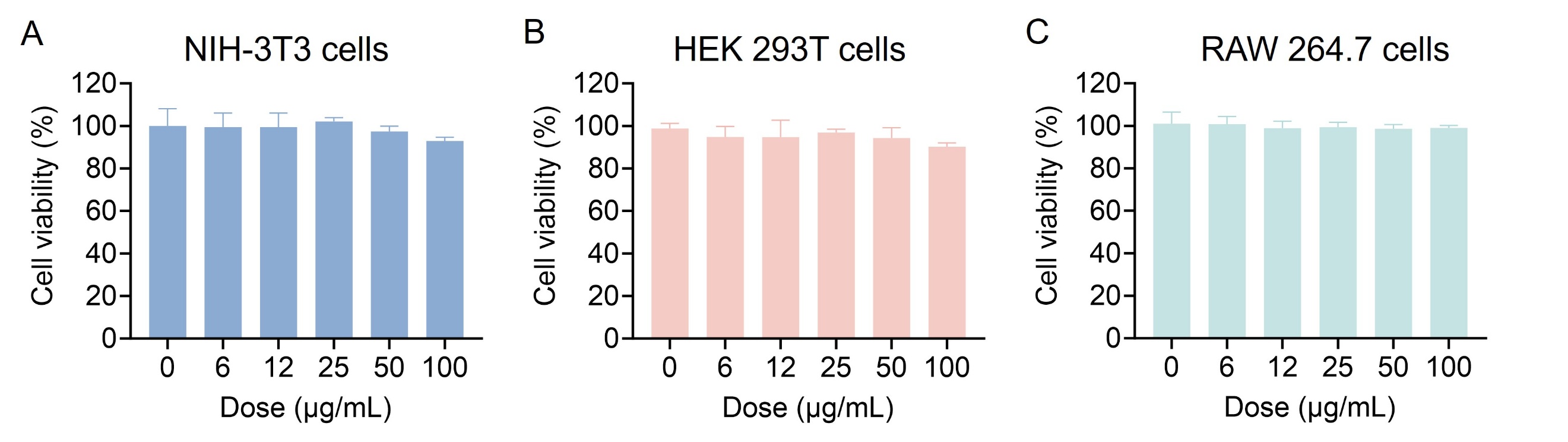


**Figure S12.** CCK-8 assay of NIH-3T3 (A), HEK 293T (B), and RAW 264.7 cells (C) after treatment with varying concentrations of CFT for 24 h (n=3).


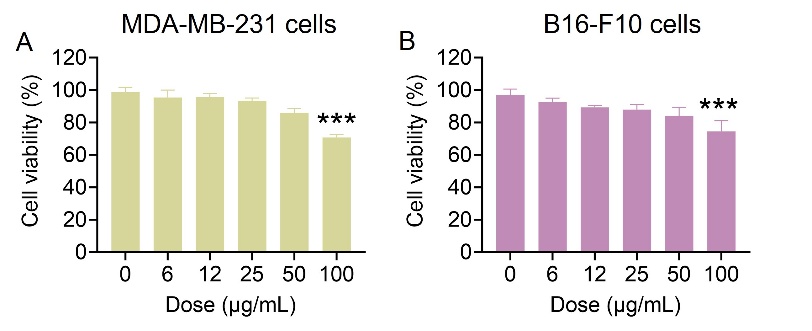


**Figure S13.** CCK-8 assay of MDA-MB-231 (A) and B16-F10 cells (B) after treatment with varying concentrations of CFT for 24 h (n=3).


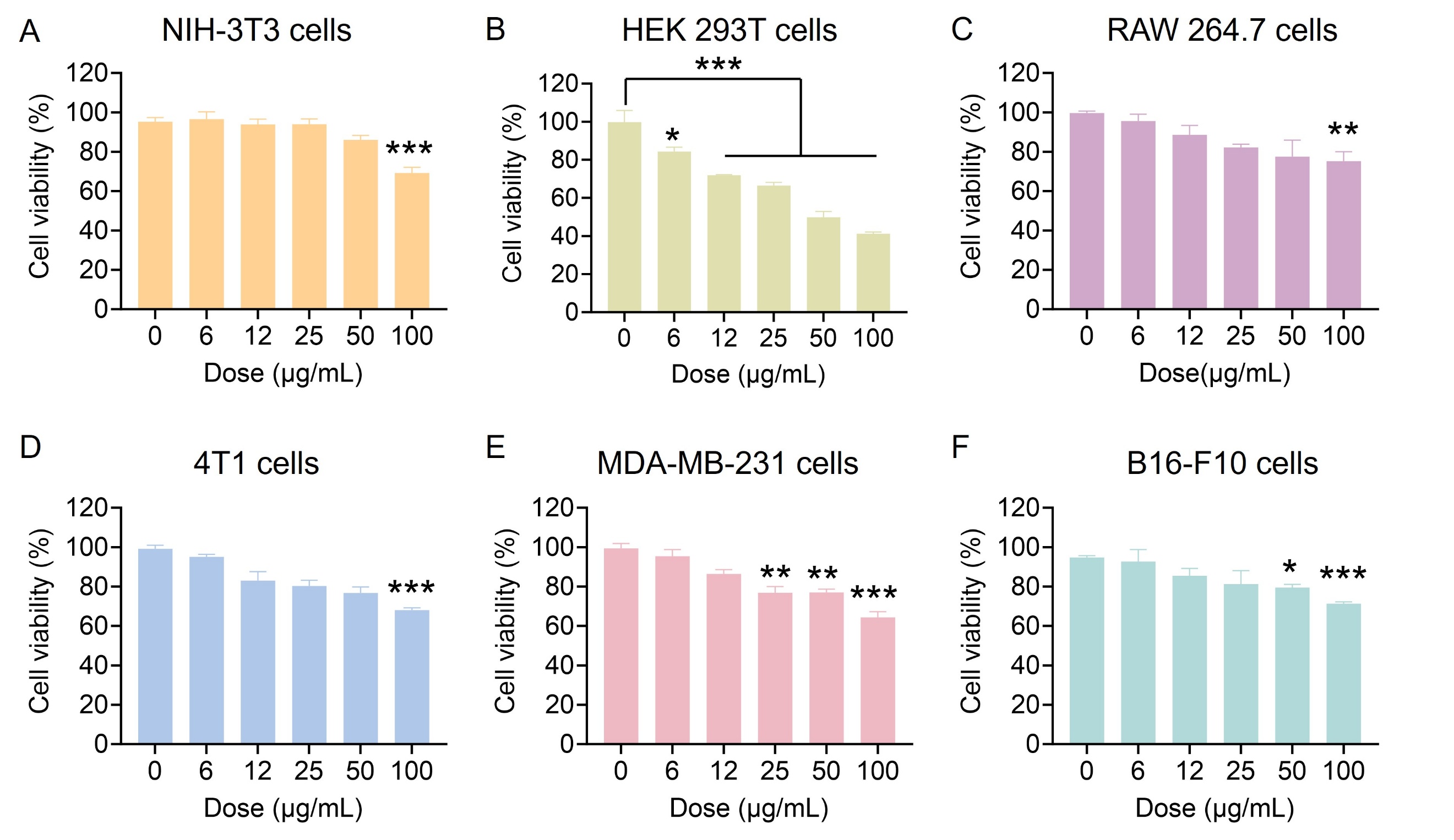
 **Figure S14.** CCK-8 assay of NIH-3T3 (A), HEK 293T (B), RAW 264.7 cells (C), 4T1 (D), MDA-MB-231 (E) and B16-F10 cells (F) after treatment with varying concentrations of CFT for 24 h at pH=6.5 (n=3).


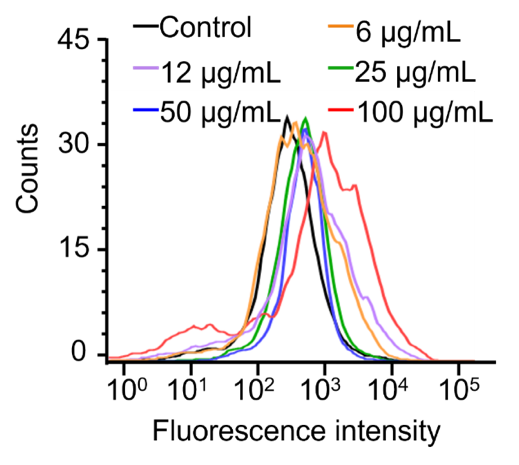


**Figure S15.** Intracellular ROS levels accessed by flow cytometry without 1064 nm laser irradiation.


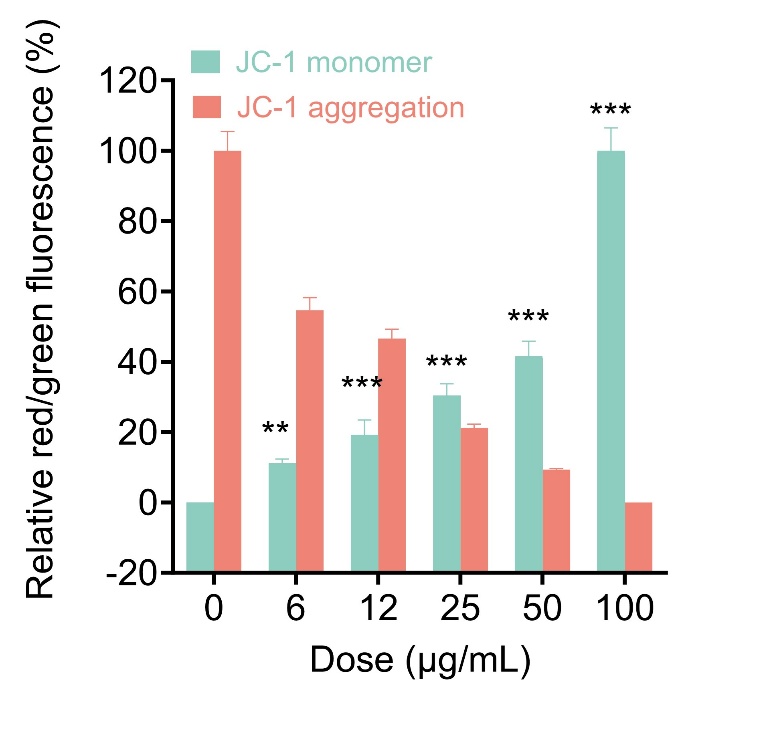


**Figure S16.** Corresponding semi-quantitative analysis of JC-1 monomer (green fluorescence) and JC-1 aggregation (red fluorescence).


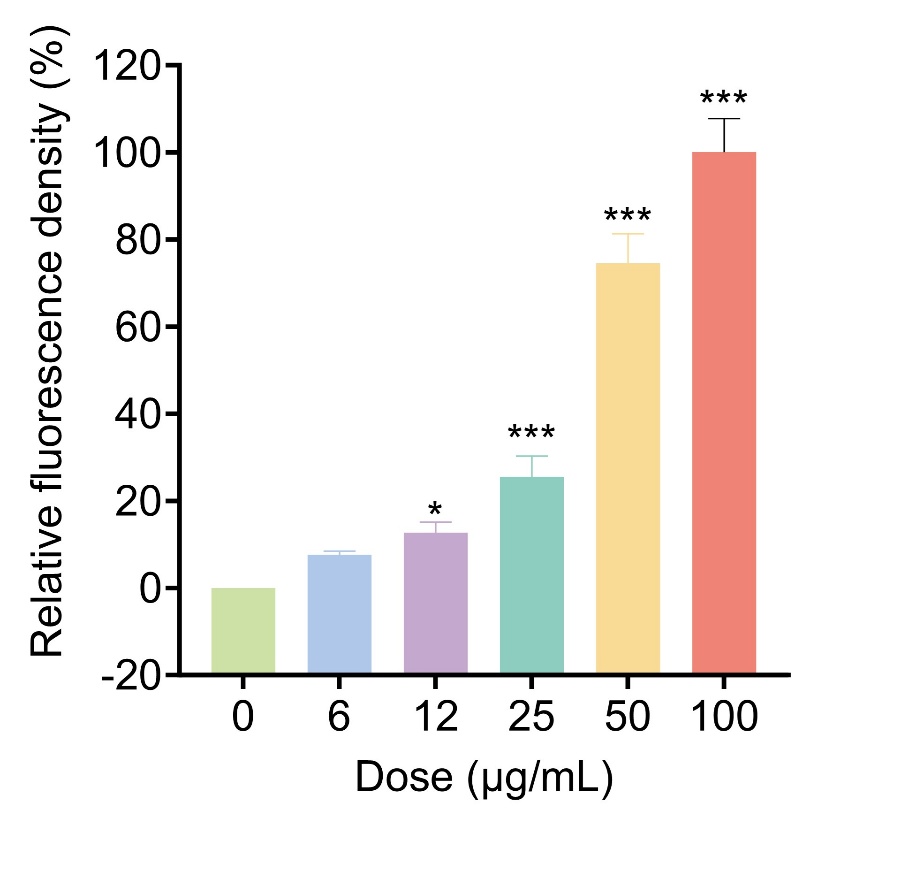


**Figure S17.** Corresponding semi-quantitative analysis of MitoSox Red (red fluorescence).


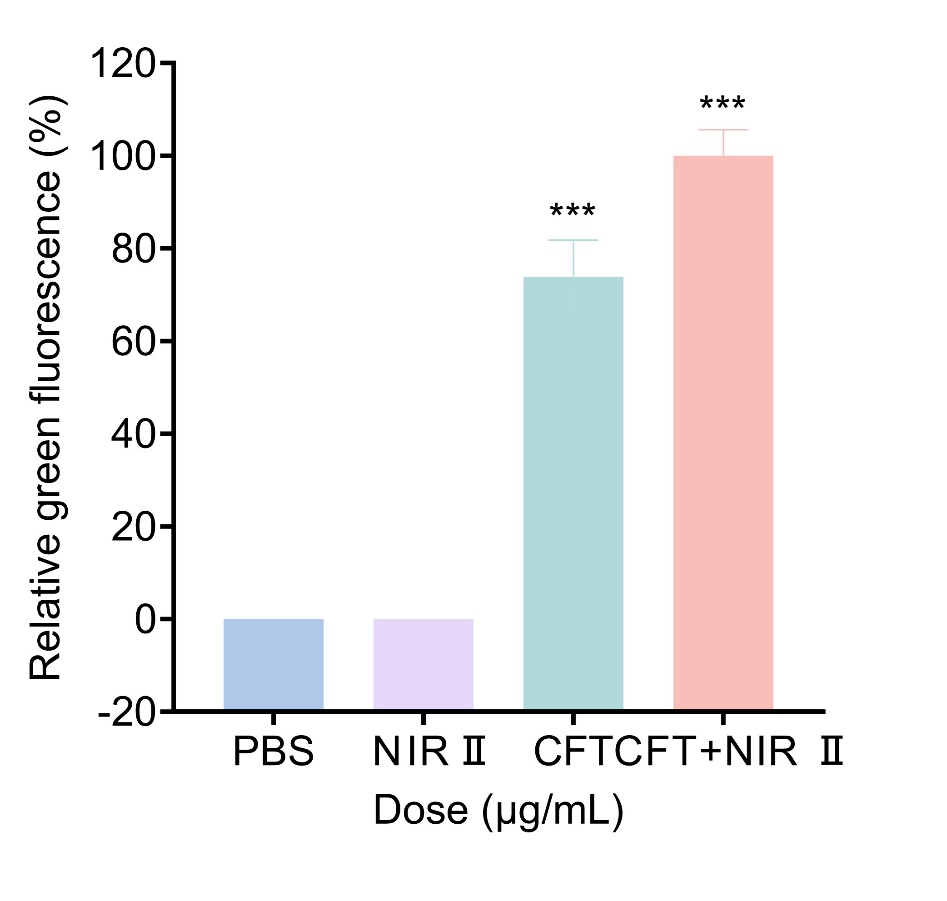


**Figure S18.** Corresponding semi-quantitative analysis of Cu^+^ BioTracker (green fluorescence).


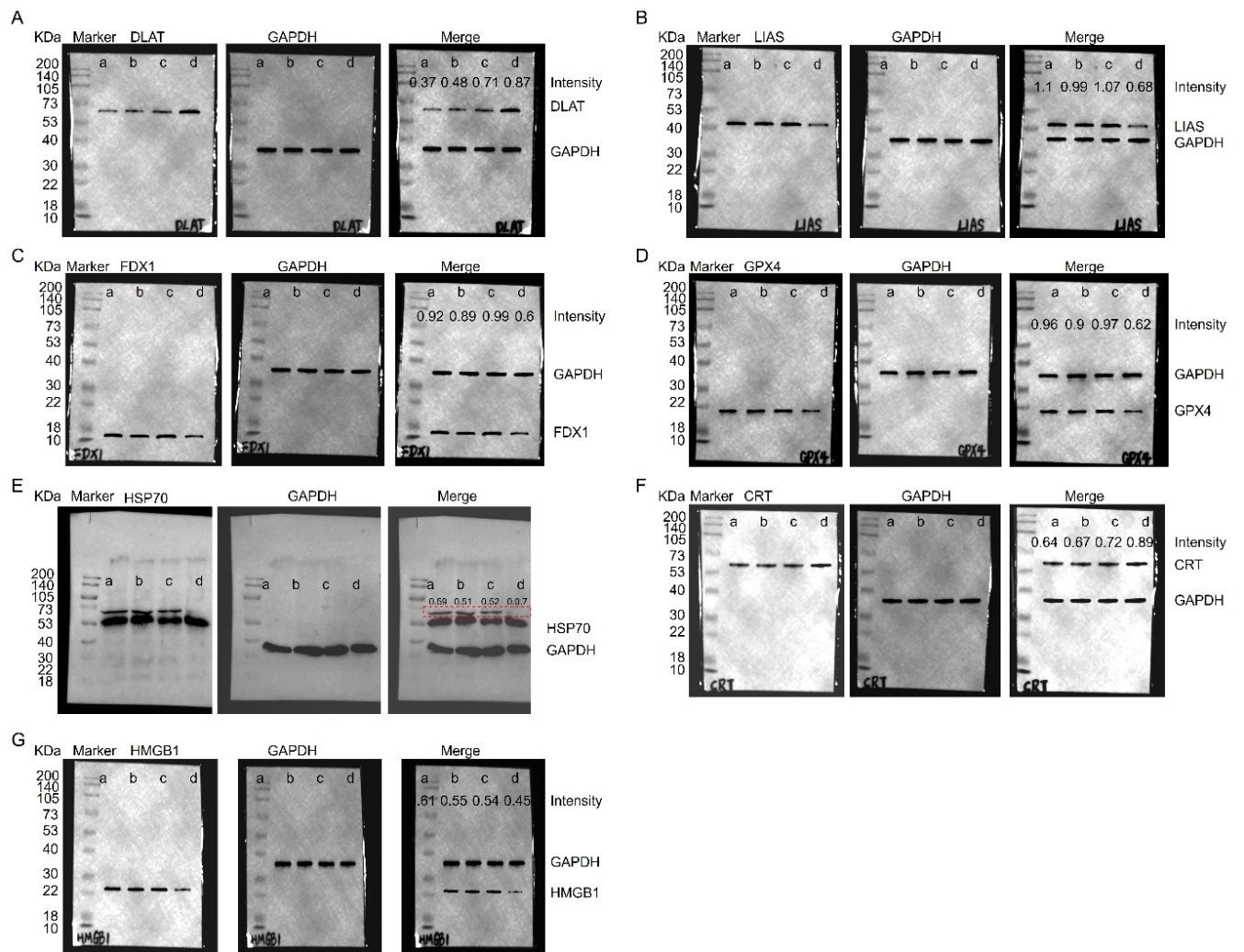


**Figure S19.** Uncropped/full-size western blots data For Figure 5B with molecular mass markers present and the lanes labelled. (A) DLAT (69 KDa). (B) LIAS (42 KDa). (C) FDX1 (13 KDa). (D) GPX4 (19 KDa). (E) HSP70 (70 KDa). (F) CRT (60 KDa). (G) HMGB1 (25 KDa), GAPDH (36 KDa). (a) PBS, (b) NIR II, (c) CFT, (d) CFT+NIR II. The intensity of protein expression was normalized to that of GAPDH using Image J software.


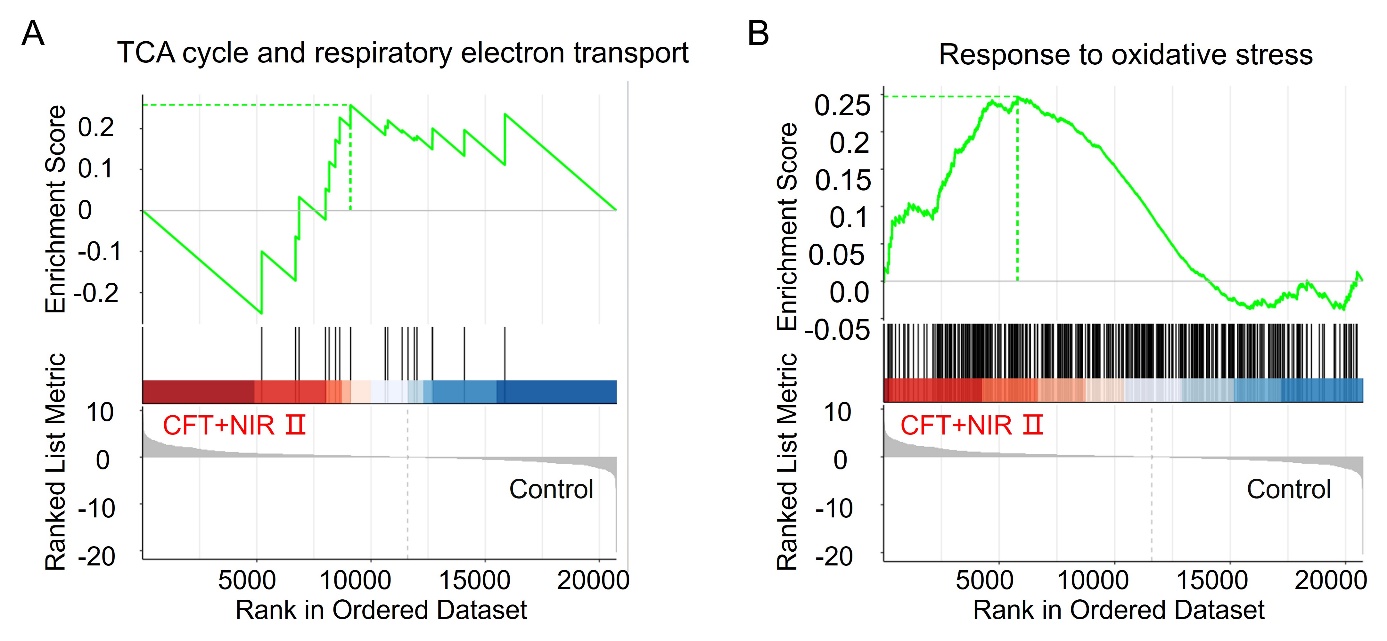


**Figure S20.** GSEA enrichment analysis of DEGs in the CFT + NIR II and control groups.


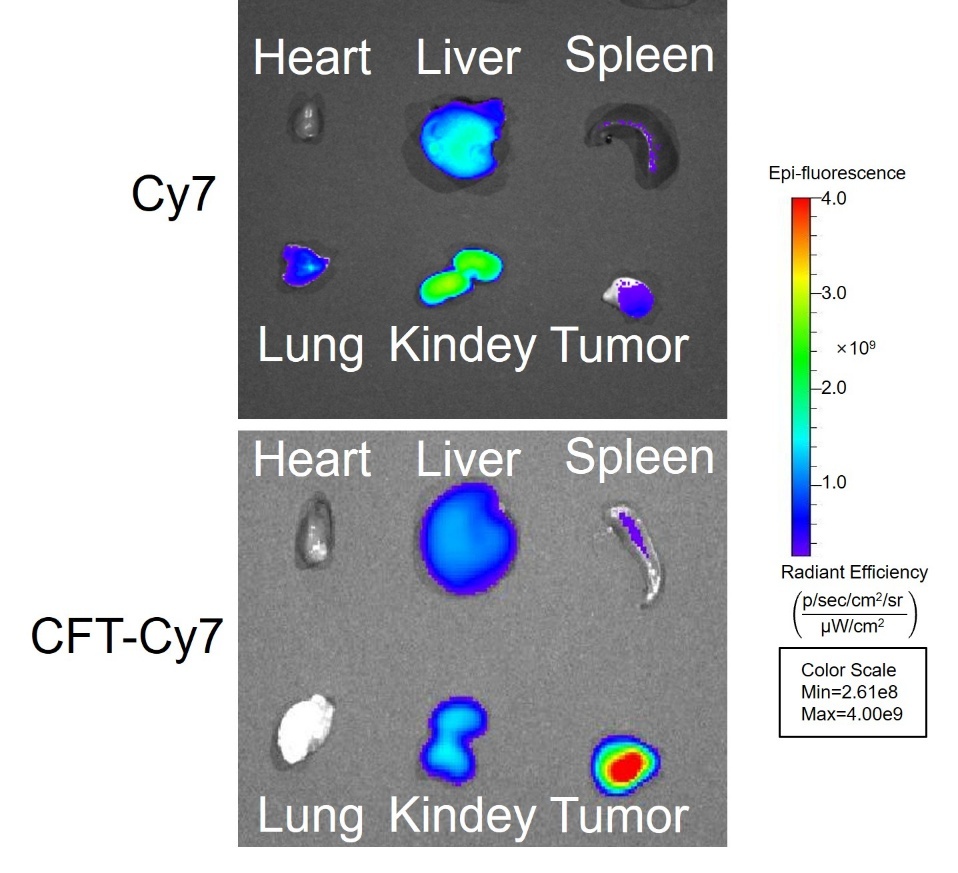


**Figure S21.** Fluorescence images of major organs (heart, liver, spleen, lung, and kidney) and tumours of mice at 24 hours post-injection of free Cy7 or CFT-Cy7.


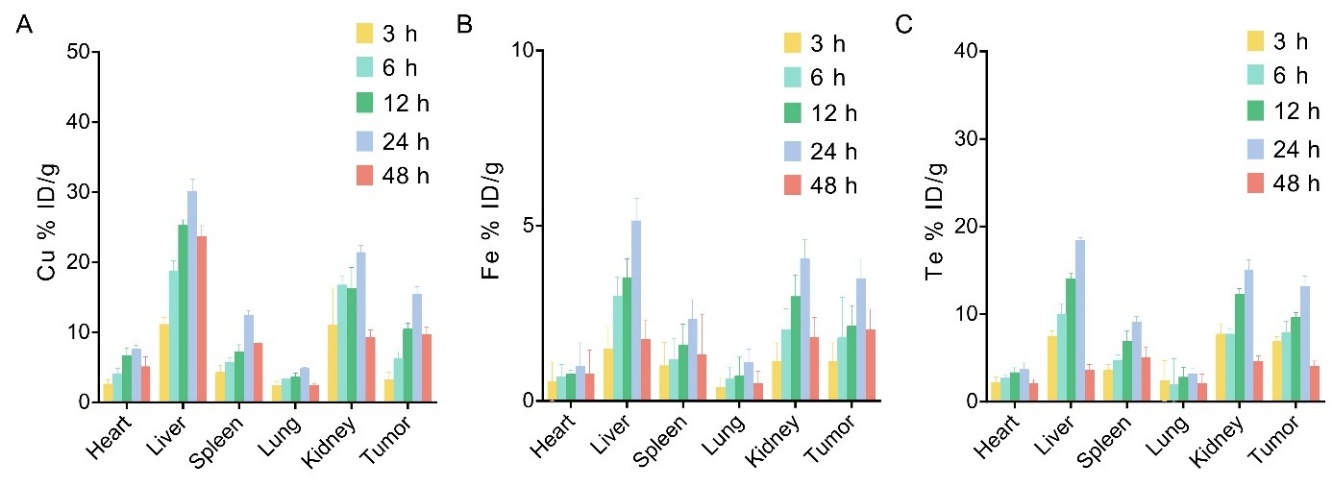
 **Figure S22.** Biodistribution of CFT in mice at 3, 6, 12 24 and 48 h postinjection, analysed by ICP-MS based on Cu element (A), Fe element (B) and Te element (C) and data were expressed as a percentage of the injected dose per gram of tissue (%ID/g) (n=3).


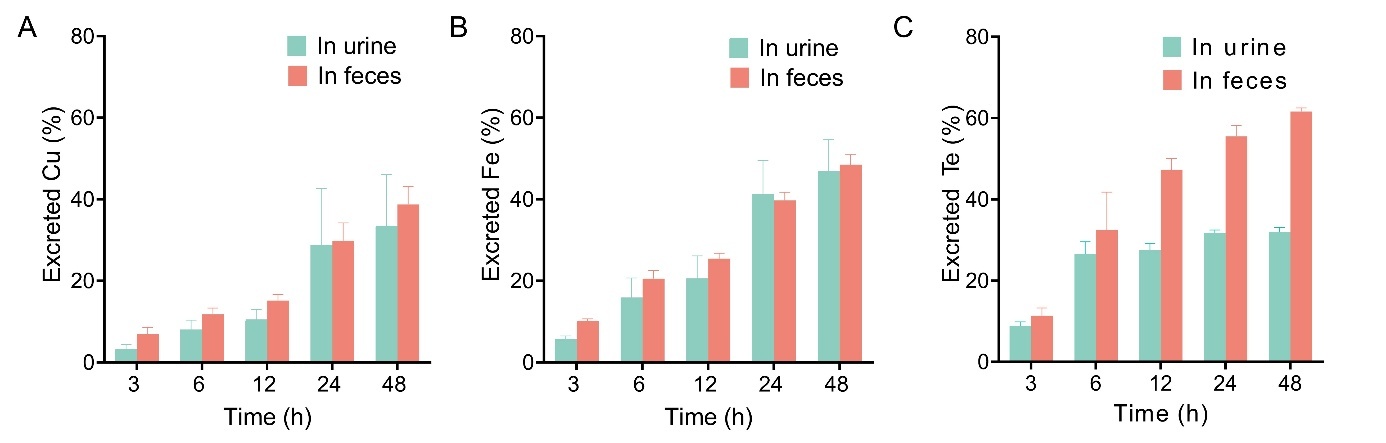
**Figure S23.** Cu, Fe and Te excretion after intravenous injection of CFT at 3, 6, 12, 24, and 48 h measured by ICP-MS (n=3).


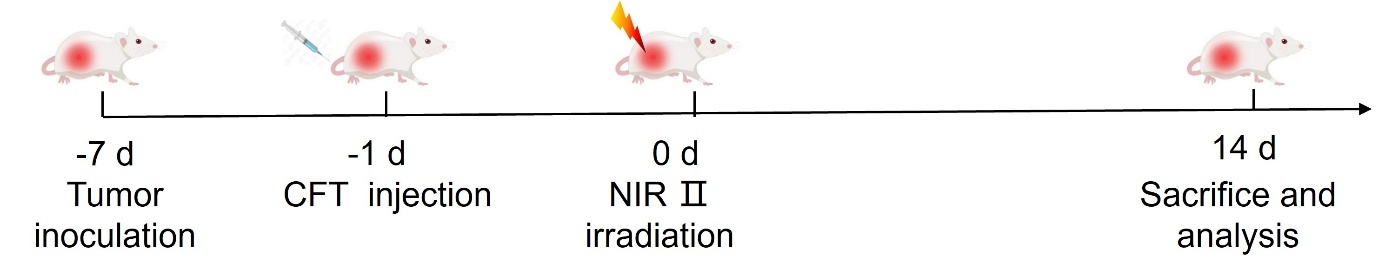


**Figure S24.** Schematic illustration of the therapeutic protocol in 4T1 tumour-bearing mice.


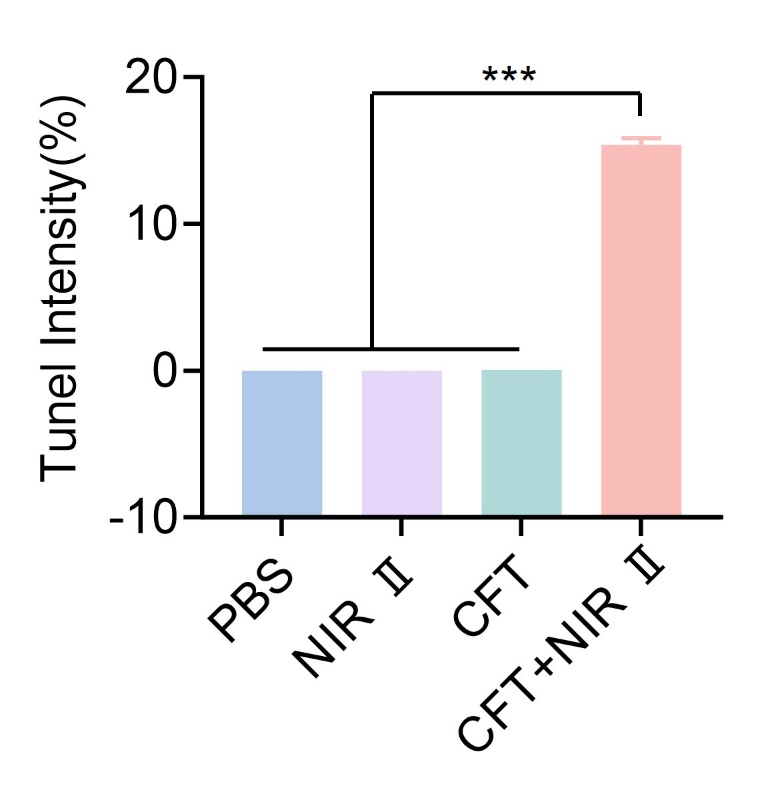


**Figure S25.** Quantitative analyses of green fluorescence intnsity of TUNEL.


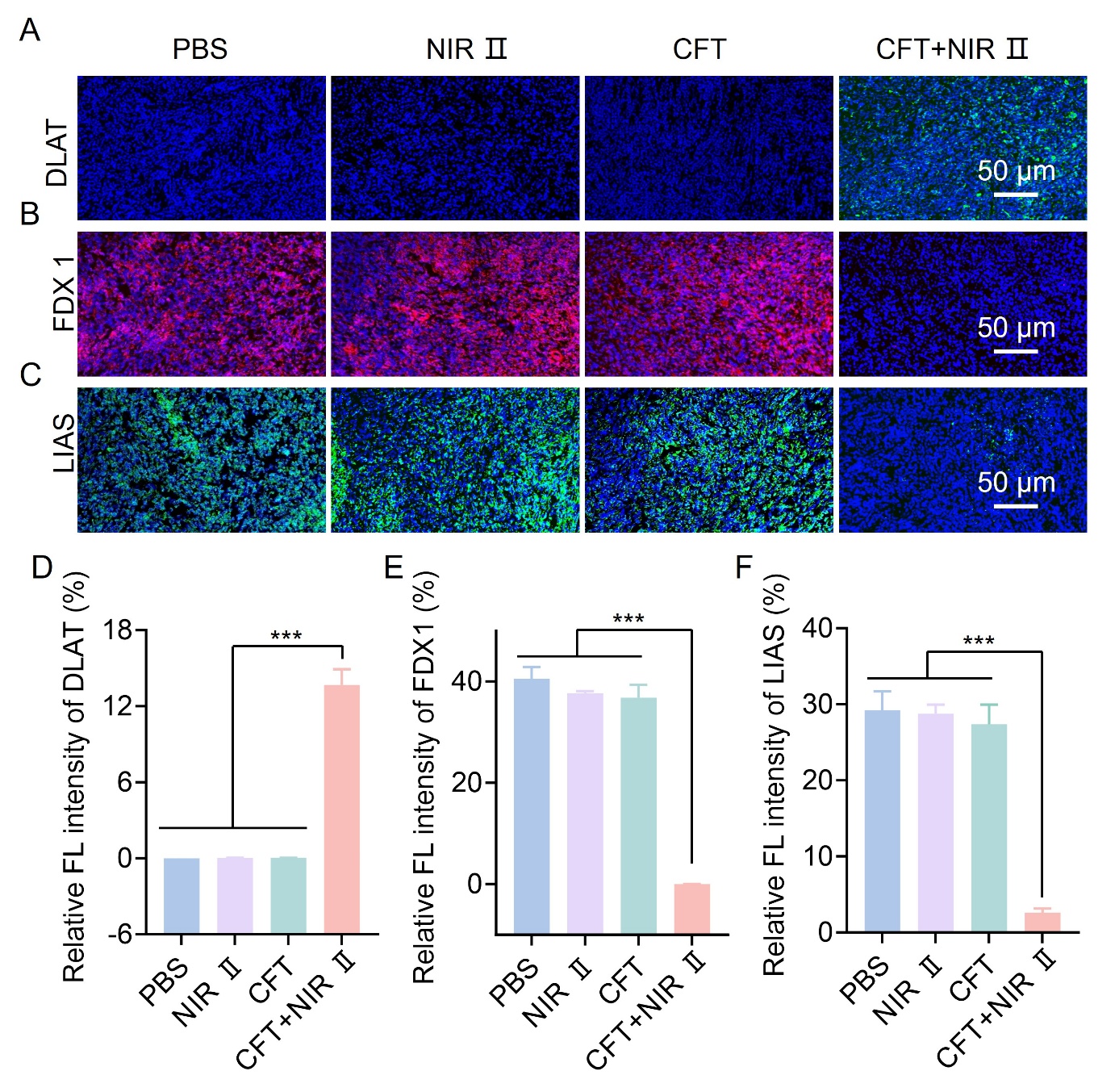


**Figure S26.** IF staining images of (A) DLAT, (B) FDX1, and (C) LIAS in tumor sections obtained at the end of treatment and quantitative analyses of (D) DLAT, (E) FDX1, and (F) LIAS.


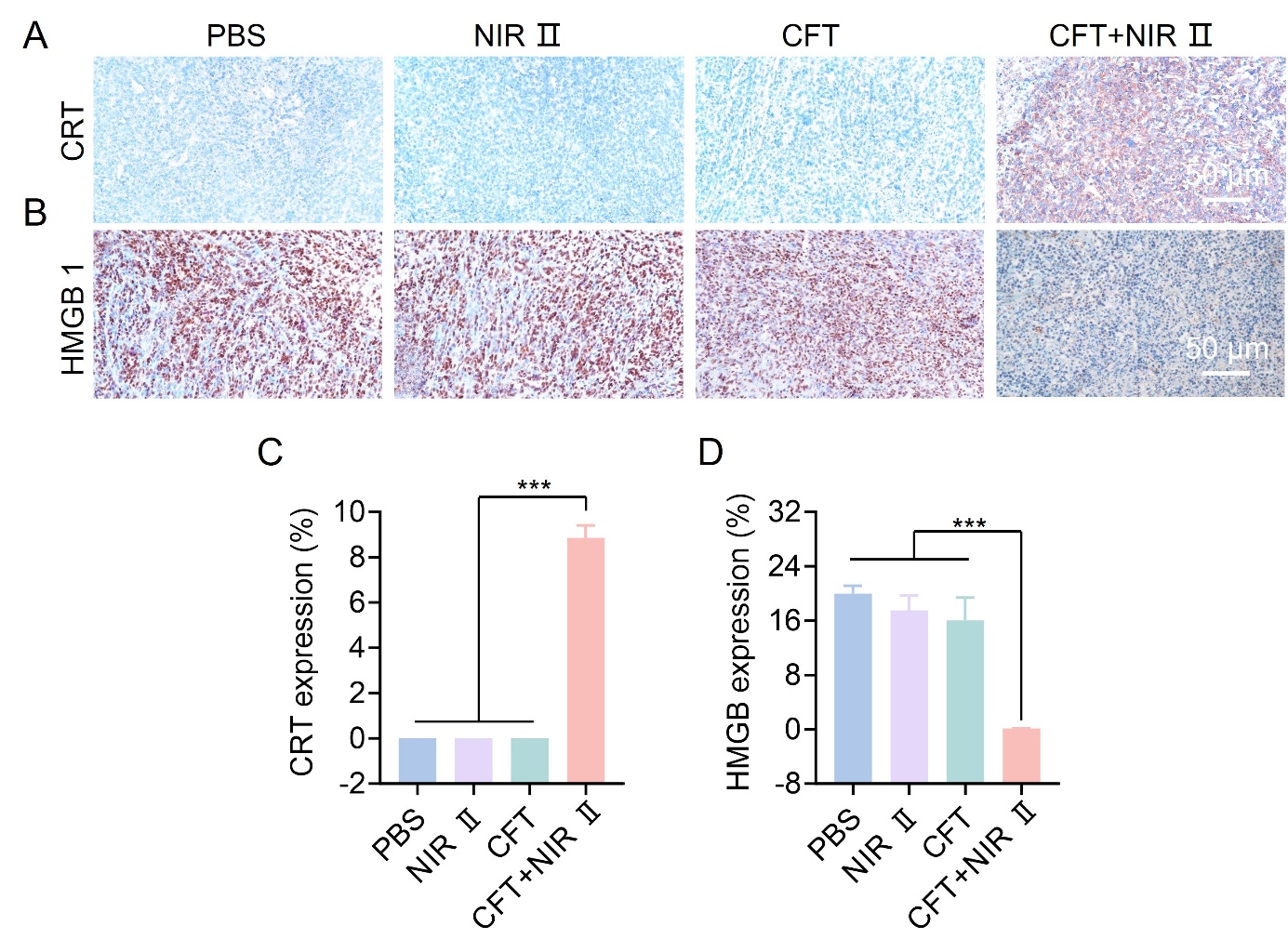


**Figure S27.** IHC staining images of (A) CRT and (B) HMGB1 in tumor sections obtained at the end of treatment and quantitative analyses of (C) CRT and (D) HMGB1.


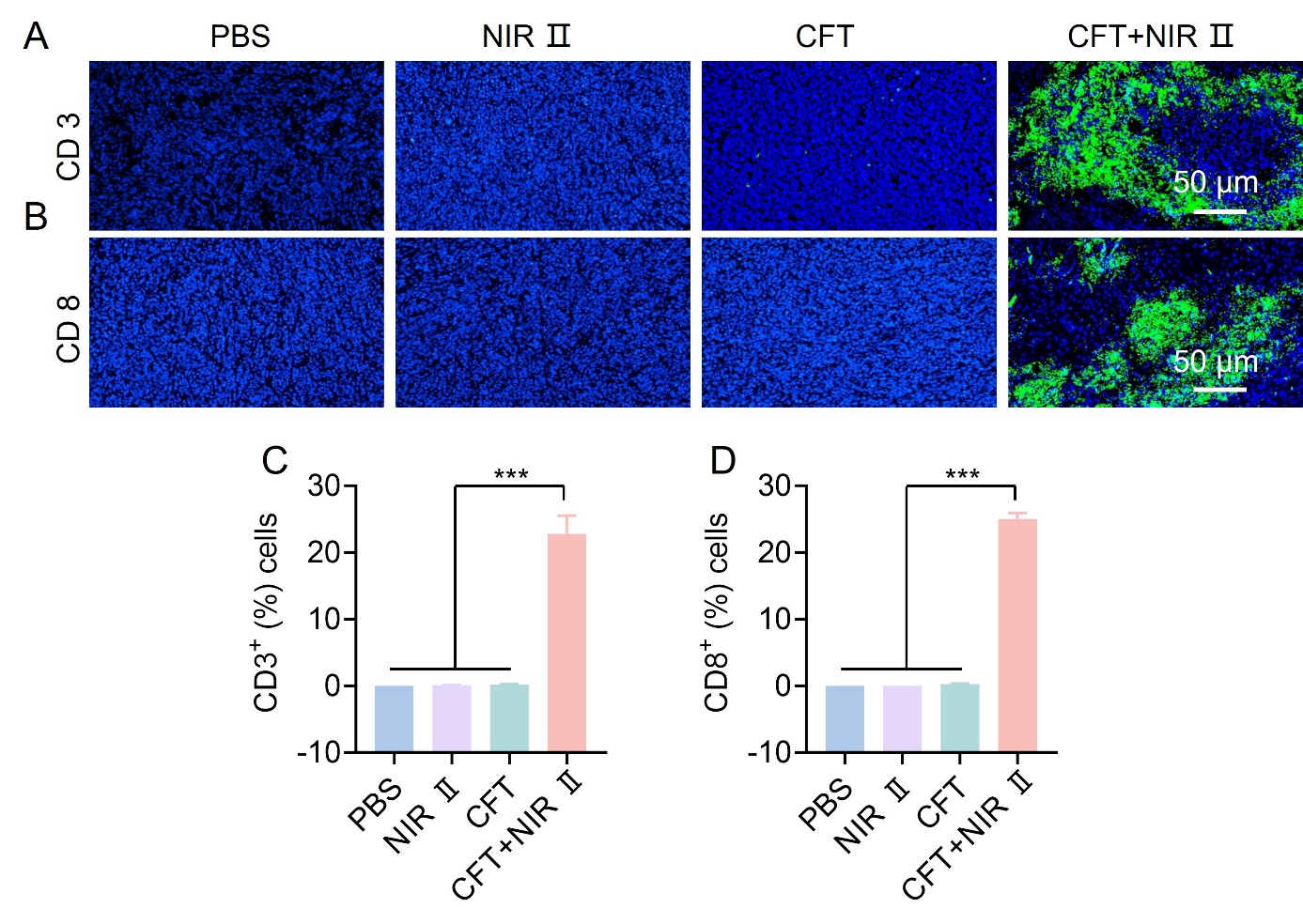


**Figure S28.** IF staining images of (A) CD^3+^ T cells and (B) CD^8+^ T cells in tumor sections obtained at the end of treatment and quantitative analyses of (C) CD^3+^ T cells and (D) CD^8+^ T cells.


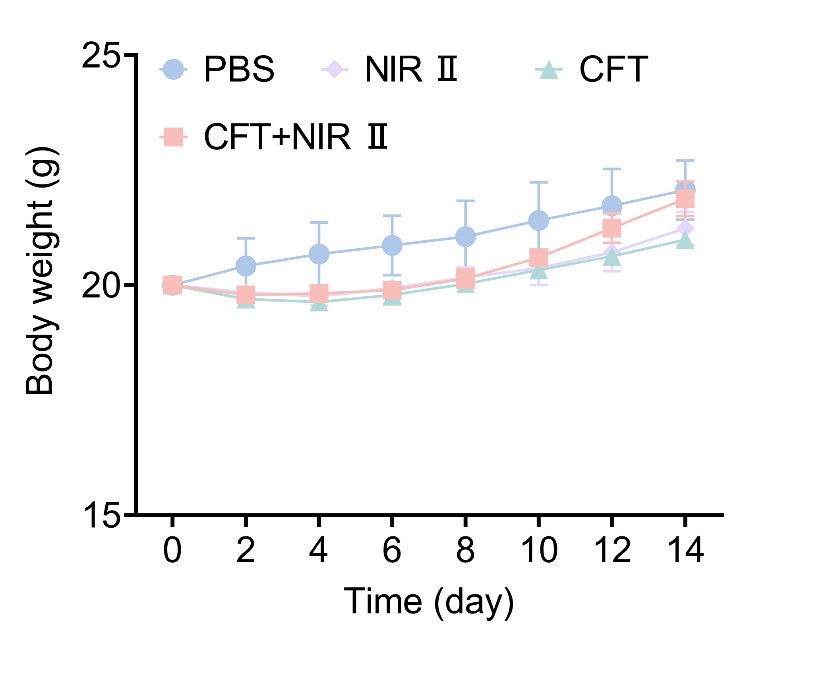


**Figure S29.** Body weight of mice from different groups for 14 days after various treatments.


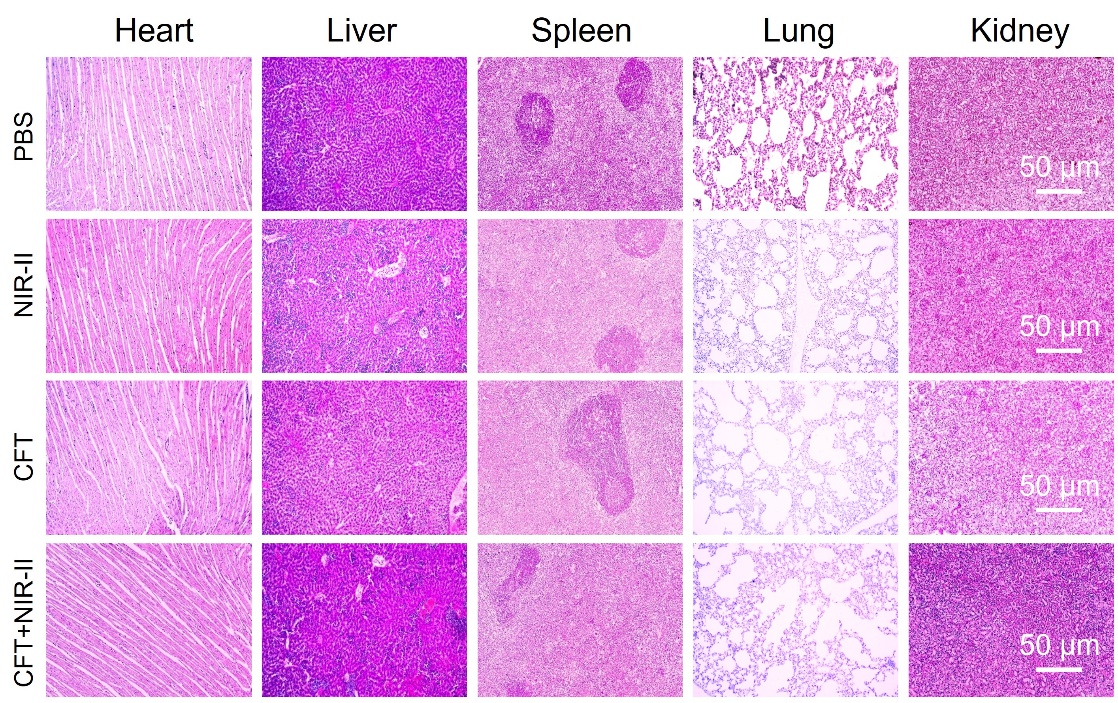


**Figure S30.** H&E stained histological images of heart, liver, spleen, lung, and kidney collected at the end of treatment.
